# Targeted deletion of *ecto-5′-nucleotidase* results in retention of inosine monophosphate content in postmortem muscle of medaka (*Oryzias latipes*)

**DOI:** 10.1038/s41598-022-22029-y

**Published:** 2022-11-03

**Authors:** Yu Murakami, Masashi Ando, Ryota Futamata, Tomohisa Horibe, Kazumitsu Ueda, Masato Kinoshita, Toru Kobayashi

**Affiliations:** 1 Department of Fisheries, Graduate School of Agriculture, Kindai University, Nakamachi 3327-204, Nara, 631-8505 Japan; 2 Division of Applied Life Sciences, Graduate School of Agriculture, Kyoto University, Kitashirakawa-Oiwake-cho, Sakyo-ku, Kyoto, 606-8502 Japan; 3 Department of Medical-Bioscience, Faculty of Bio-Science, Nagahama Institute of Bio-Science and Technology, 1266 Tamura-cho, Nagahama, Shiga 526-0829 Japan; 4 Institute for Integrated Cell-Material Sciences (WPI-iCeMS), KUIAS, Kyoto University, Kyoto, 606-8501 Japan; 5 Division of Applied Biosciences, Graduate School of Agriculture, Kyoto University, Kitashirakawa-Oiwake-cho, Sakyo-ku, Kyoto, 606-8502 Japan

**Keywords:** Biotechnology, Genetics

## Abstract

Inosine monophosphate (IMP) is an important indicator of meat freshness and contributes to its umami taste. An attractive strategy for enhancing umami is to suppress the IMP-degrading activity and increase the IMP content in the skeletal muscle through genome editing technology using the CRISPR-Cas9 system. However, the molecular mechanisms underlying IMP degradation remain unclear. We cloned two *ecto-5′-nucleotidase* genes, designated as *ecto-5′-nucleotidase*-*a* (*nt5ea*) and *ecto-5′-nucleotidase*-*b* (*nt5eb*), from medaka (*Oryzias latipes*), a vertebrate model organism. Expression analysis using embryos showed that *nt5ea* or *nt5eb* overexpression remarkably upregulated IMP degradation, and that the IMP-degrading activity was higher in Nt5ea than in Nt5eb. Furthermore, we established frame-shifted or large deletion (lacking *nt5ea* or *nt5eb* locus) mutant strains and assayed the effects of gene disruptions on the amount of IMP in skeletal muscle. The *nt5ea*-deficient medaka showed considerable higher levels of IMP at 48 h postmortem than did the wild-type fish. The *nt5eb* mutants also exhibited higher IMP contents than that in the wild types, but the increase was less than that in the *nt5ea* mutants. Our results demonstrated that *nt5e* is an important regulator of IMP levels in skeletal muscle and that its loss of function was effective in maintaining IMP content.

## Introduction

Fishes are a valuable source of protein for humans, and also provide a large variety of vitamins, minerals, and lipids^1^. The worldwide demand for fishery products has increased over the past few decades owing to population growth and increased health awareness^2^. Aquaculture holds great potential as a sustainable solution to meet the ever-increasing demand for fish; thus, it is one of the fastest growing global food sectors. The contribution of aquaculture to global fisheries increased from 26.0% in 2000 to 46.1% in 2018^3^. Despite the rapid growth of aquaculture, most cultured fish species are either still sourced from the wild or in the earlier stages of breeding; this is different to many crops and livestock, which have been subjected to selective breeding for several millennia^4–6^. Some fish strains have been cultivated using several biotechnologies such as the chemical mutagenesis and transgenics; however, genetic improvements have been limited by the generation interval, laborious work of screening mutants, or the emergence of unpredictable traits^7–9^.

Genome editing using the CRISPR-Cas9 system offers new opportunities and solutions for breeding a wide range of organisms. In contrast to conventional random mutagenesis and transgenesis, this system induces targeted and flexible alterations in the chromosomal sequences^10^. The co-delivery of the endonuclease Cas9 combined with single guide RNA (sgRNA) into cells generates DNA double-strand breaks (DSBs) at the target loci, consequently stimulating endogenous repair pathways such as non-homologous end joining (NHEJ)^11^. NHEJ efficiently yields small nucleotide insertions or deletions (indels), leading to frameshift mutations and premature stop codons resulting in gene disruption^12^. The CRISPR-Cas-based approach has been applied to more than 10 species of cultured fish and has expedited their molecular breeding; however, only a few typical traits such as growth enhancement, disease resistance, and sterility, have currently been identified^13–15^. This situation is partly due to the fact that genetic dissection in aquatic organisms lags behind those of mammals such as humans and mice. To accelerate further genetic improvements by the CRISPR-Cas9 system in fish, the identification of new target genes responsible for commercial traits is needed.

The value of food is defined by various aspects such as safety, nutrition, and taste. Umami is a key factor in determining the palatability and acceptability of foods and has been widely recognized as the fifth basic taste, along with the other four basic tastes: sweet, sour, salty, and bitter^16^. Inosine monophosphate (IMP) is an important umami substance in fish that accumulates in postmortem muscle through the degradation of adenosine triphosphate (ATP) as follows: ATP → adenosine diphosphate (ADP) → adenosine monophosphate (AMP) → IMP → inosine (HxR) → hypoxanthine (Hx)^17^. The degradation of ATP to IMP progresses rapidly after death, whereas IMP is gradually broken down into non-taste components such as HxR and Hx^18^. This process is conserved among various fish species and livestock, and the amount of IMP in the muscle is an indicator of meat quality and freshness^19,20^.

The suppression of enzymes with the IMP-degrading activity is an attractive strategy to maintain the umami taste in fish. Enzymes belonging to the 5′-nucleotidase (Nt5) family catalyze the hydrolytic dephosphorylation of nucleoside monophosphates to nucleosides and orthophosphate^21^. In humans, seven different Nt5 activities have been characterized with regard to substrate specificity, cellular distribution, oligomerization state, and molecular size^22^. One of them, ecto-5′-nucleotidase (Nt5e), has a broad substrate specificity for both ribo- and deoxyribonucleoside monophosphates^23^. Enzyme kinetic data, mainly from mammals, have shown that the *K*_m_ value (IMP) of Nt5e is approximately 100 folds smaller than that of other Nt5 subtypes, suggesting its primary role in the degradation of IMP during ATP catabolism^21^. Thus, *nt5e* knockout fish are expected to have decreased IMP-degrading activity and accumulate IMP in their meats, leading to enhanced umami. To generate the desired mutants using the CRISPR-Cas9 system, it is necessary to understand the functions of the target genes and identify their DNA sequences in advance. However, basic knowledge about Nt5e in fish remains lacking. In previous studies, IMP-degrading activity has been detected in crude extracts from tissues such as the skeletal muscle, liver, and kidney in several fish species based on biochemical approaches^24,25^. Although these studies helped to characterize the properties of IMP degradation as a whole, our insight into the function of Nt5e itself is limited. A previous study using zebrafish (*Danio rerio*) reported that Nt5e extracted from brain membranes hydrolyzed AMP, uridine monophosphate (UMP), guanosine monophosphate (GMP), and cytidine monophosphate (CMP)^26^; however, there is still no clear evidence that Nt5e is active against IMP in teleosts. Thus, it is crucial to determine whether Nt5e catalyzes the IMP degradation in fish, which is the key to generating umami-enhanced fish by *nt5e* knockout.

To establish *nt5e*-deficient strains, we employed two types of knockout approaches: single sgRNA-directed mutagenesis and dual sgRNA-directed large gene deletion. These methods have both advantages and disadvantages. The former method can potentially be applied for food production by generating mutants with small indels that may occur in nature. In the field of fisheries, new breeds with several nucleotide deletions have been established in tiger puffer (*Takifugu rubripes*) and red sea bream (*Pagrus major*) using the CRISPR-Cas9 system, and have been officially approved as edible in Japan^27–29^. However, the former method does not necessarily cause loss of function of the target genes. If genome editing tools generate nonsense mutations within open reading frames, translation may be initiated from an in-frame ATG other than the authentic translation initiation codon^30^. This phenomenon, known as illegitimate translation (ITL), may result in the expression of functional proteins from edited genes harboring small indels. In contrast, the latter method with dual sgRNAs can avoid such ITL and ensure the loss of function. The simultaneous cleavage of two target sites induces a large-scale genomic deletion of up to approximately 100 kbp^31^, allowing for the removal of the target whole coding sequence (CDS). However, large-scale defects are unusual in nature, unlike small indels^32^, making it difficult to accept large-deleted organisms as food items. Considering the food applicability and knockout reliability, we used these two methods to produce *nt5e*-deficient fish.

In this study, we used medaka (*Oryzias latipes*) because of features such as daily spawning, rapid sexual maturity, and suitability for genome editing techniques, allowing for simple genetic analyses. We first cloned two *nt5e* genes in medaka to examine their conservation among multiple fish species using in silico analyses. Next, by injecting the RNA that was synthesized in vitro into the embryos, we overexpressed each Nt5e fused with reporter proteins, such as enhanced green fluorescent protein (EGFP) and firefly luciferase (LUC). With the embryo extracts injected with RNAs, we evaluated the IMP degradation activity of each *nt5e*. Finally, by disrupting each *nt5e* using the CRISPR-Cas9 system, the effects of genetic mutations on the IMP levels in the skeletal muscle were investigated. To assess whether the mutants exhibited any other phenotypic abnormalities, we also measured standard length and body weight and conducted microscopic examination of their organs.

## Results

### Identification and characterization of two nt5e genes in medaka

**Figure 1:**
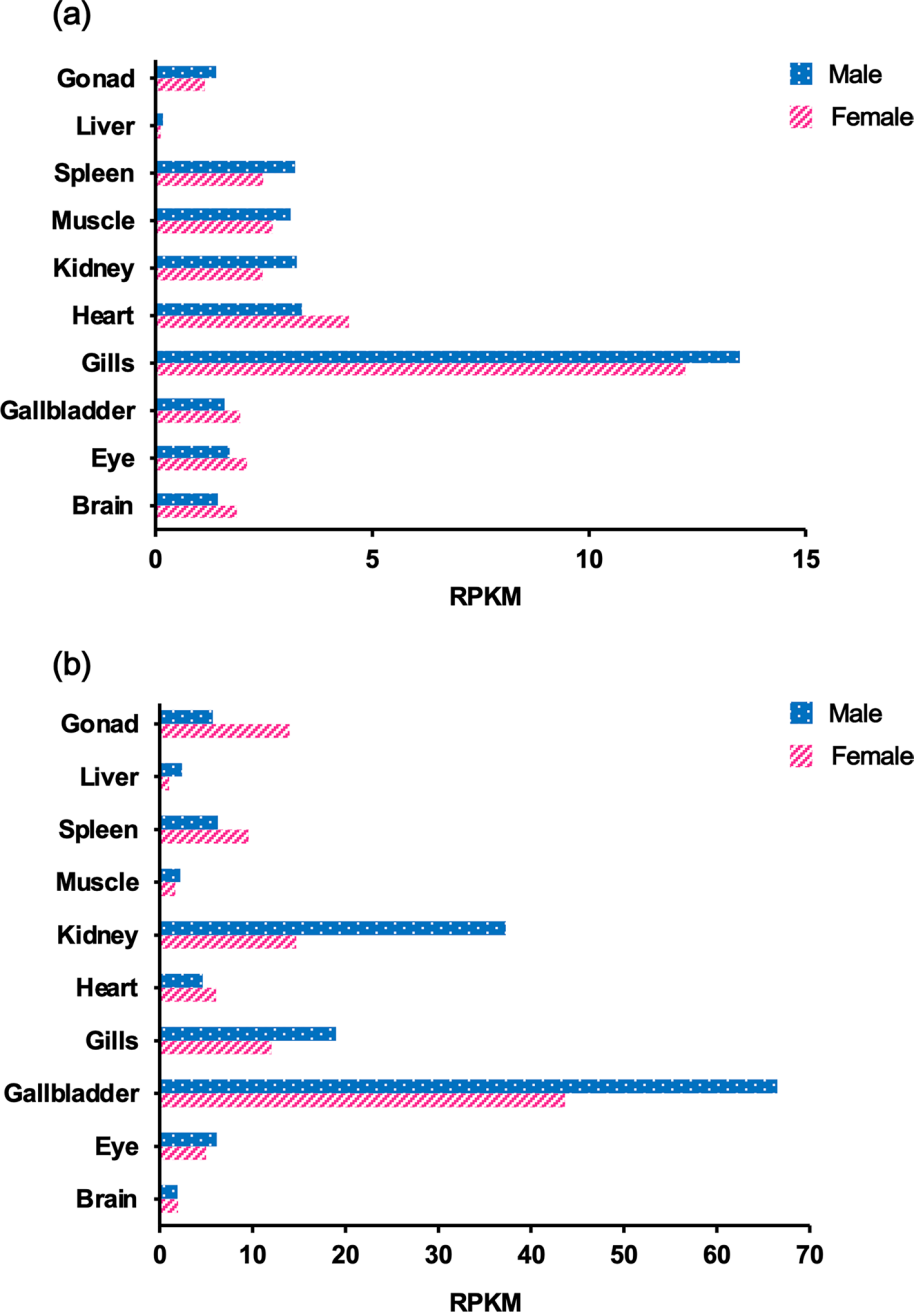
RNA sequencing analyses for *nt5ea* (**a**) and *nt5eb* (**b**) genes in various organs of the male and female Cab strain medaka. To normalize individual differences, the total RNA of each organ was extracted from 8 to 16 fish. Transcription expression values were estimated as reads per kilobase of exon per million mapped sequence reads (RPKM).

**Figure 2:**
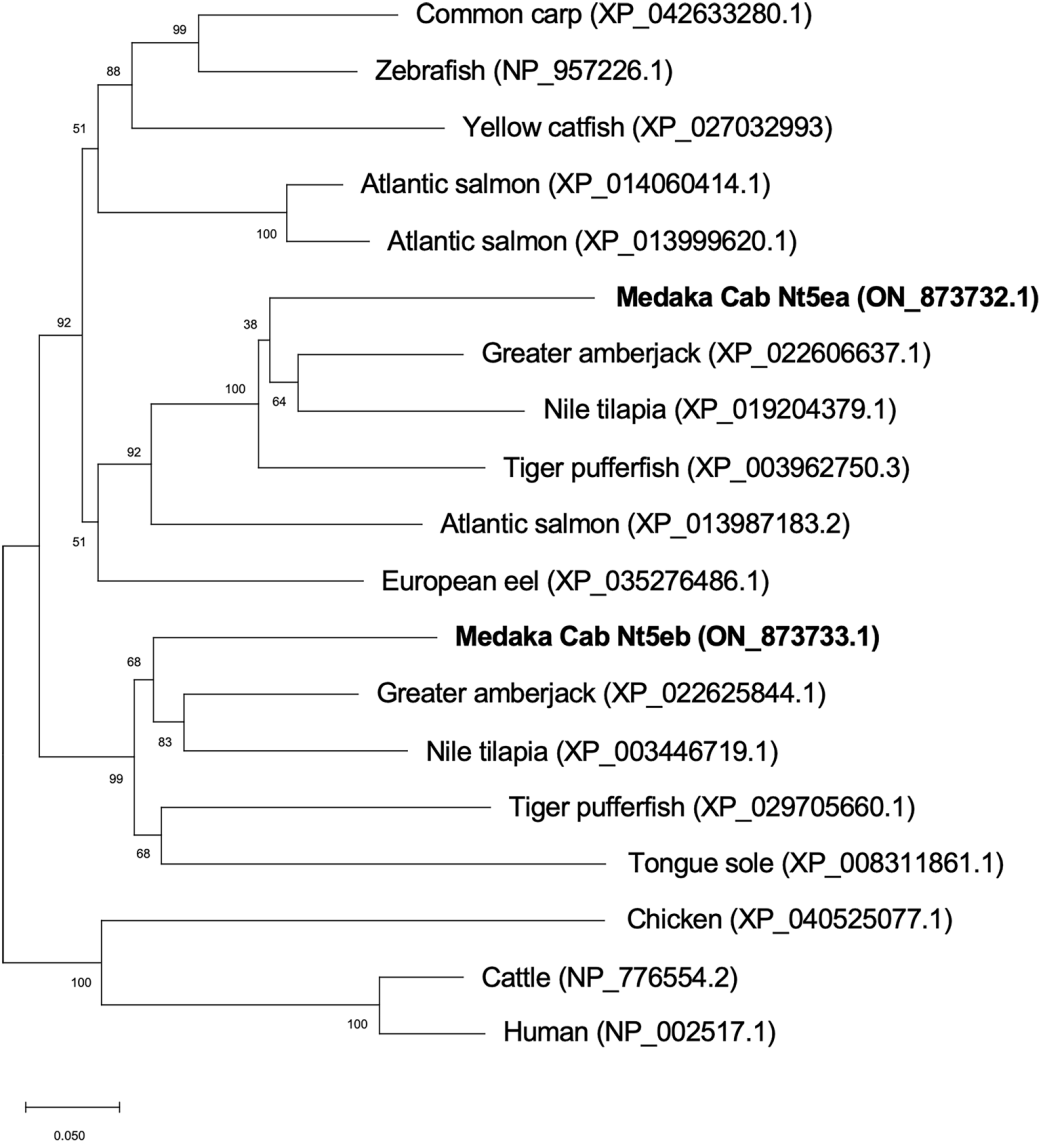
Phylogenetic analysis of Nt5e amino acid sequences. The scale bar shows the substitution rate per residue. Numbers at nodes indicate boot-strap values, as percentages, obtained from 1000 replicates. The gene accession number for each species is provided along with the species name.

To clone *nt5e* genes from the Japanese medaka Cab strain, we searched the NCBI genome database and identified two types of *nt5e* in the Japanese medaka Hd-rR strain as zebrafish *nt5e* orthologs. Thus, we termed these two *nt5e* genes *nt5ea* (Hd-rR: GenBank accession number LOC101174549) and *nt5eb* (Hd-rR: GenBank accession number LOC101168262). RNA-seq analysis of the Cab strain showed that the expression levels of *nt5ea* and *nt5eb* were the highest in the male gill and gallbladder, respectively (Fig. 1a,b). Using cDNA from the male gill or gallbladder, the CDSs of *nt5ea* or *nt5eb* were isolated by reverse transcription–polymerase chain reaction (RT-PCR), and then cloned into each plasmid with reporter genes (Fig. S1a,b). The full-length open reading frames of Cab *nt5ea* and *nt5eb* cDNA comprised of 1731 bp encoding 577 amino acid (AA) residues and 1743 bp encoding 581 AA residues, respectively. The nucleotide sequences of *nt5ea* and *nt5eb* in the medaka Cab strain mostly corresponded to those of the Hd-rR strain, but several mutations were detected. Five silent mutations were identified in the nucleotide sequence of *nt5ea*, whereas two silent mutations and one missense mutation were found in *nt5eb* (Fig. S2a,b). Our in silico analysis using SignalP identified the possible secretion signal peptides in the first 28 AA (^1^MTLRWRCCALGALLGLLLRLDSWSGASG^28^) of Nt5ea and the first 31 AA (^1^MDAVRPERSAAQLLRFCPVLLILSGPCMTAA^31^) of Nt5eb (Fig. S3a,b). Sequence comparison revealed that Cab Nt5ea and Nt5eb shared the highest AA identity (73.7% and 74.0%) and similarity (81.3% and 81.5%) with their counterparts of Greater amberjack (*Seriola dumerili*) (Table S1). As shown in Fig. 2, phylogenetic analysis revealed that all fish Nt5e sequences formed a cluster separated from mammalian Nt5e. Within the fish clade, Cab Nt5ea and Nt5eb clustered together with several other Nt5e sequences from Perciformes fish, such as *S. dumerili* and Nile tilapia (*Oreochromis niloticus*).

### Comparison of IMP-degrading activity between Nt5ea and Nt5eb

**Figure 3:**
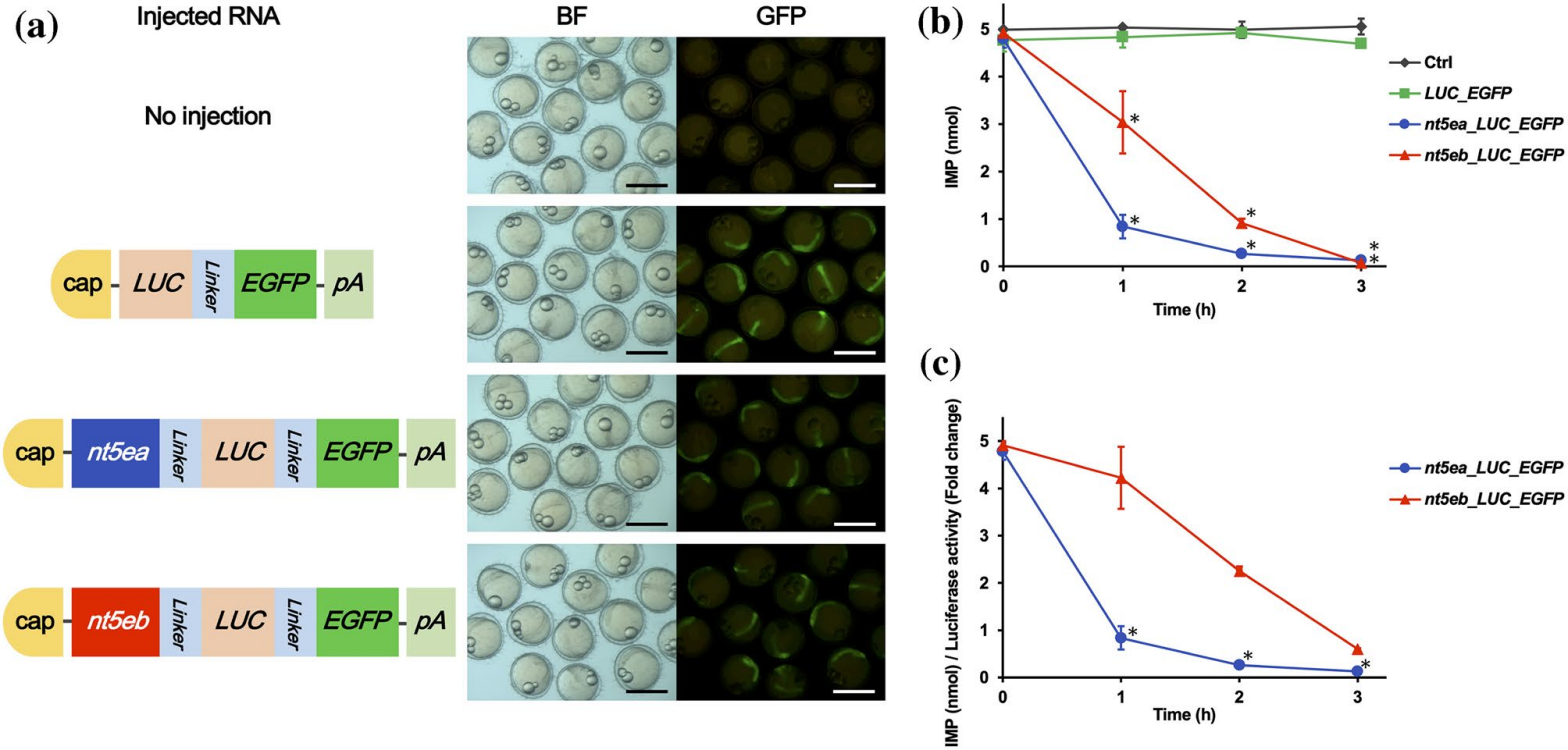
Assay of inosine monophosphate (IMP)-degrading activities of Nt5ea and Nt5eb. (**a**) Representative GFP expression in embryos at 1 d post fertilization. Green fluorescence was detected from embryos injected with RNA encoding *LUC*-*EGFP*, *nt5ea*-*LUC*-*EGFP*, or *nt5eb*-*LUC*-*EGFP*, but not from embryos without microinjection. *BF* bright field, *GFP* green fluorescent protein, scale bar 1 mm. (**b**) IMP degradation via overexpression of *nt5ea* and *nt5eb*. Embryos without microinjection were used as a control (Ctrl). Data are presented as the mean ± SD of three determinations with different enzyme preparations. The asterisks indicate that the values are significantly different to those of the Ctrl group according to one-way ANOVA followed by Tukey’s HSD test. *P < 0.05. (**c**) Comparison of IMP-degrading activities between Nt5ea and Nt5eb after normalization with LUC luminescence. The asterisks indicate that the values of the group *nt5ea*-*LUC*-*EGFP* were significantly less than those of the group *nt5eb*-*LUC*-*EGFP* according to one-way ANOVA followed by Tukey’s HSD test. *P < 0.05.

To validate the IMP-degrading activities of Cab Nt5ea and Nt5eb, in vitro transcribed RNAs (Fig. 3a) were injected into the embryos, leading to overexpression of the enzymes. Using the embryos with green fluorescence (Fig. 3a), we prepared the reaction mixtures and assayed their reactivity to IMP. As shown in Fig. 3b, the amount of IMP in the group injected with RNA of *nt5ea*-*LUC*-*EGFP* or *nt5eb*-*LUC*-*EGFP* decreased more quickly in a time-dependent manner compared to that in the group injected with *LUC*-*EGFP* and the control (uninjected) group. These results show that both N5ea and Nt5eb catalyzed the degradation of IMP. Furthermore, there was no significant difference in the value of IMP at all time points examined between the group injected with *LUC*-*EGFP* and the control group, indicating that the fusion protein of LUC-EGFP did not show reactivity to IMP (Fig. 3b). To accurately compare the IMP-degrading activities of Nt5ea and Nt5eb, we normalized the amount of IMP to the value of LUC luminescence (Fig. S4). The comparison revealed that the group injected with *nt5ea*-*LUC*-*EGFP* degraded IMP more rapidly than the group injected with *nt5eb*-*LUC*-*EGFP* (Fig. 3c). This result showed that Nt5ea had a stronger enzymatic activity against IMP than Nt5eb. Because IMP was not detected in medaka embryos at 1 d post-fertilization (dpf) (data not shown), our assay was not biased by endogenous IMP.

### Establishment of complete nt5e disrupted strains

To generate *nt5ea-* or *nt5eb-*deficient mutants, four sgRNAs (sgRNA-nt5ea-1, sgRNA-nt5ea-2, sgRNA-nt5eb-1, and sgRNA-nt5eb-2) were designed on the first or last exon of each target locus (Fig. S6c,g). A heteroduplex mobility assay (HMA) analysis confirmed that these sgRNAs possessed DSB-inducing activities (Fig. S5a,b), which were used in the following two knockout methods. First, to obtain mutants with a frame-shifted genome, the complex of Cas9 protein with sgRNA-nt5ea-1 or sgRNA-nt5eb-1 targeted for each first exon was injected into embryos (Fig. S6a,e). This approach successfully produced a 2- and 10-bp deletions in the first exons of *nt5ea* and *nt5eb*, respectively (Fig. S6b,f), which were predicted to induce a non-functional truncated protein (Fig. S7a,b). Second, to generate mutants with large deletions at the target loci, the complex of the Cas9 protein with each pair of sgRNAs (sgRNA-nt5ea-1/sgRNA-nt5ea-2 or sgRNA-nt5eb-1/sgRNA-nt5eb-2) was introduced into the embryos (Fig. S6c,g). This approach yielded a mutant fish with 5521- and 10,900-bp deletions at the *nt5ea* and *nt5eb* loci, respectively, which lacked almost the entire CDS region (Fig. S6c,d,g,h). The results of the microinjections and screening of knockout founders are summarized in Supplementary Table 2. The genotype of each fish, comprising wild type, heterozygotes, or homozygotes, was confirmed by the PCR-based analyses. These genotyped fish were used for subsequent analyses.

### Effects of nt5e knockout on ATP-related compounds in postmortem muscle

**Figure 4:**
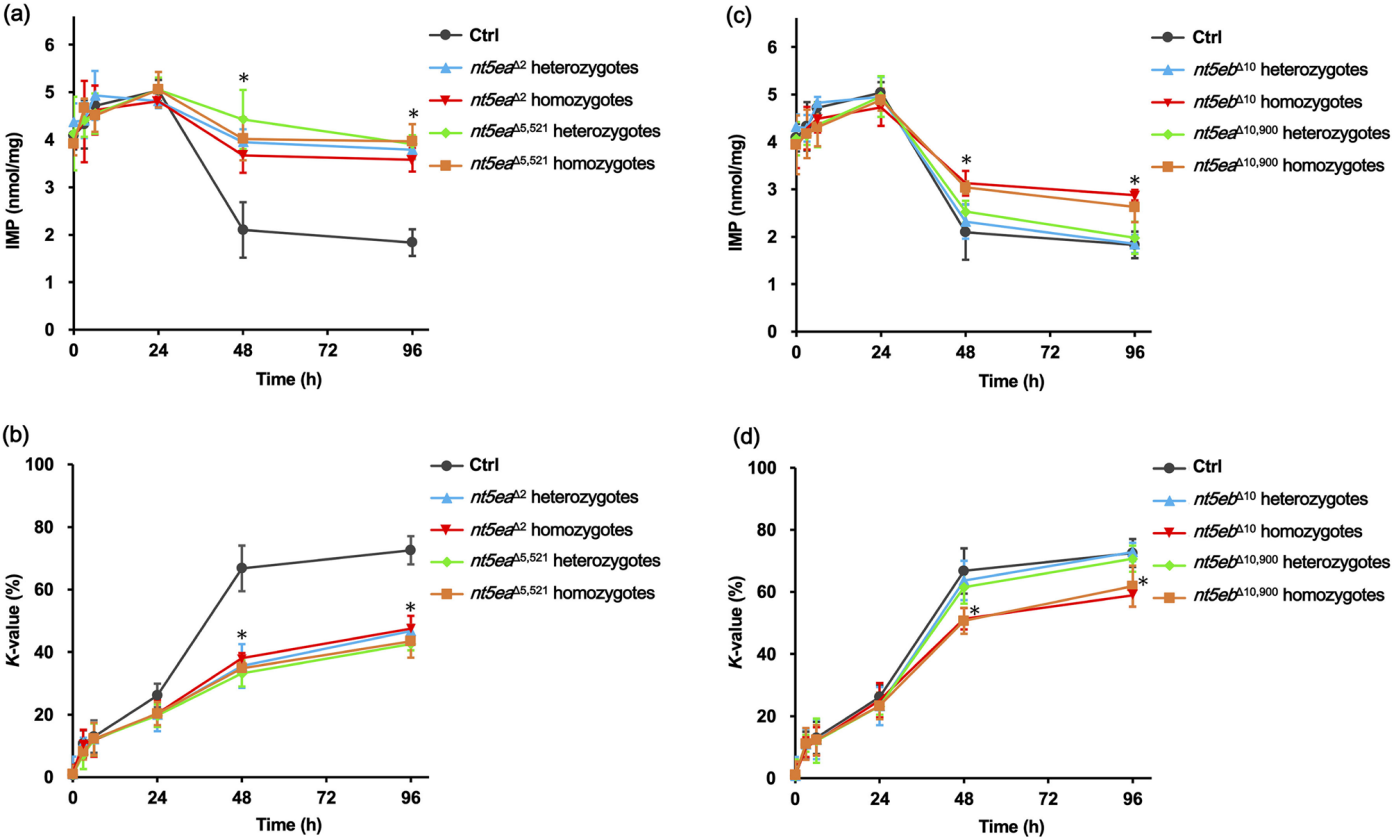
Chemical evaluation of fish meat stored in refrigeration. (**a**,**b**) Inosine monophosphate (IMP) contents and *K*-values of wild-type and *nt5ea* mutant fish from 0 to 96 h postmortem. Data are presented as the mean ± SD (n = 4/group at 0, 3, 6, and 24 h and n = 7/group at 48 and 96 h). Wild-type fish harboring no mutation were used as a control (Ctrl). The asterisks indicate that the values of each mutant group, *nt5ea*^+/∆2^, *nt5ea*^∆2/∆2^, *nt5ea*^+/∆5,521^, *nt5ea*^∆5,521/∆5,521^, were significantly different compared to those of the Ctrl group according to one-way ANOVA followed by Tukey’s HSD test, *P < 0.05. (**c**,**d**) IMP contents and *K*-values of wild-type and *nt5eb* mutant fish from 0 to 96 h postmortem. Data are presented as the mean ± SD (n = 4/group at 0, 3, 6, and 24 h and n = 7/group at 48 and 96 h). Wild-type fish harboring no mutation were used as a control (Ctrl). The asterisks indicate that the values of each homozygous mutant group, *nt5eb*^∆10/∆10^ or *nt5eb*^∆10,900/∆10,900^, were significantly different compared to those of the Ctrl group according to one-way ANOVA followed by Tukey’s HSD test. *P < 0.05.

To evaluate the effects of *nt5ea* or *nt5eb* gene disruption on the IMP levels and *K*-values in the muscles, high-performance liquid chromatography (HPLC)-based quantitative analysis was performed. As shown in Fig. 4a, the IMP contents in both heterozygous groups (*nt5ea*^+/∆2^: 3.79 ± 0.20 and *nt5ea*^+/∆5,521^: 3.91 ± 0.19) and homozygous groups (*nt5ea*^∆2/∆2^: 3.58 ± 0.25 and *nt5ea*^∆5,521/∆5,521^: 3.96 ± 0.36) were significantly higher than those in the control group (*nt5ea*^+/+^: 1.83 ± 0.28) at 96 h postmortem. The IMP level in each mutant group was also significantly higher than that in the control group at 48 h postmortem, although there was no significant difference from 0 to 24 h (Fig. 4a). As shown in Fig. 4b, the *K*-values in the *nt5ea*-mutated groups, including both heterozygotes (*nt5ea*^+/∆2^: 46.73 ± 2.03 and *nt5ea*^+/∆5,521^: 42.58 ± 2.01) and homozygotes (*nt5ea*^∆2/∆2^: 47.51 ± 4.09 and *nt5ea*^∆5,521/∆5,521^: 43.46 ± 5.25), were significantly lower than those in the control group (*nt5ea*^+/+^: 72.56 ± 4.51) at 96 h postmortem. The *K*-value of each mutant group was also significantly lower than that of the control group at 48 h postmortem, although there was no significant difference from 0 to 24 h (Fig. 4b). These results showed that the knockout of *nt5ea* was useful in preventing an increase in the *K*-value at 48 h postmortem. Since there was no significant difference between the heterozygotes and homozygotes in both the IMP level and *K*-value for every sampling point, the homozygous and heterozygous mutations at the *nt5ea* locus were equally effective for the inhibition of elevated *K*-values. Subsequently, *nt5eb* mutants were subjected to the measurement of ATP-related compounds using HPLC. As shown in Fig. 4c, the homozygotes harboring 10 bp- or 10,900 bp-deletion at the *nt5eb* locus exhibited significant larger amounts of IMP (*nt5eb*^∆10/∆10^: 2.88 ± 0.34 and *nt5eb*^∆10,900/∆10,900^: 2.63 ± 0.17) than those of the control (*nt5eb*^+/+^: 1.83 ± 0.28) at 96 h postmortem. The IMP levels of both homozygous strains (*nt5eb*^∆10/∆10^ and *nt5eb*^∆10,900/∆10,900^) at 48 h postmortem were also significantly higher than that of the control; however, there was no difference from 0 to 24 h. By contrast, there was no significant difference in the IMP contents between the control and heterozygous groups (*nt5eb*^+/∆10^ or *nt5eb*^+/∆10,900^) at every assaying point (Fig. 4c). As shown in Fig. 4d, the *K*-values of homozygotes (*nt5eb*^∆10/∆10^: 58.92 ± 3.65 and *nt5eb*^∆10,900/∆10,900^: 61.88 ± 6.59) were significantly lower than those of the control (*nt5eb*^+/+^: 72.56 ± 4.51) at 96 h postmortem. The *K*-values of both homozygous strains (*nt5eb*^∆10/∆10^ and *nt5eb*^∆10,900/∆10,900^) at 48 h postmortem were also significantly lower than those of the control group, although there was no significant difference from 0 to 24 h. In contrast, there was no significant difference in the *K*-value between the control and the heterozygous groups (*nt5eb*^+/∆10^ or *nt5eb*^+/∆10,900^) at any measurement point (Fig. 4d). These results showed that the homozygous mutations at the *nt5eb* locus helped maintain the *K*-value, but the heterozygous mutations at the *nt5eb* locus did not yield such effects. Thus, the quantitative results for *nt5eb* knockout were in contrast to those for *nt5ea* knockout, which was effective in both heterozygous and homozygous mutants.

### Morphological and histological analysis of nt5e-deficient fish

**Figure 5:**
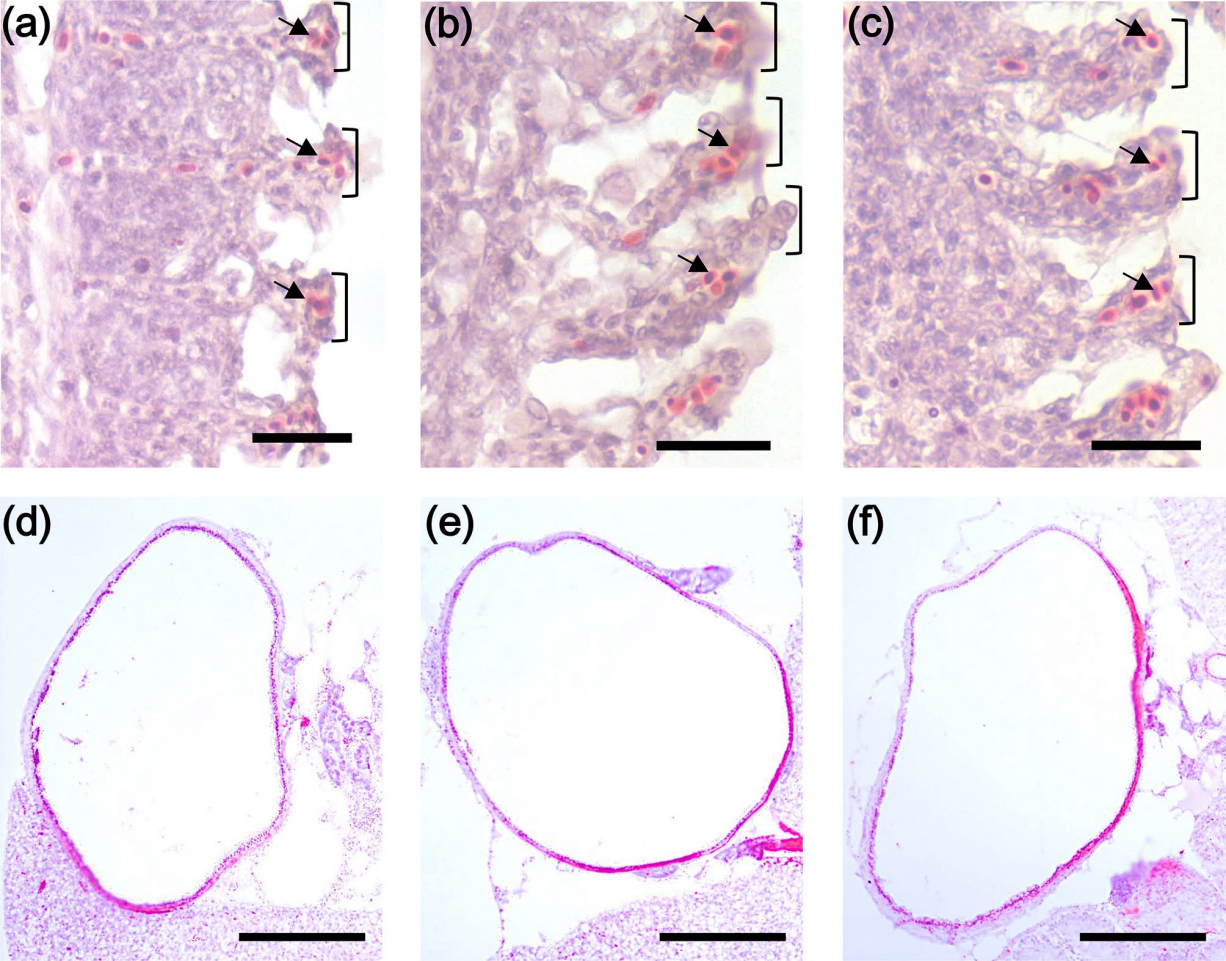
Histological evaluation of adult *nt5e* mutants. Transverse gill section of wild-type (**a**), *nt5ea*^+/∆2^ heterozygous (**b**), and *nt5ea*^∆2/∆2^ homozygous (**c**) fish at 12 weeks post-hatching. Black parentheses and arrows show lamellae and erythrocytes, respectively. Transverse gallbladder section of wild-type (**d**), *nt5eb*^+/∆10^ heterozygous (**e**), and *nt5eb*^∆10/∆10^ homozygous (**f**) fish at 12 weeks post-hatching. Scale bars indicate 25 µM (**a**–**c**) and 200 µM (**d**–**f**), respectively.

To examine the effects of *nt5e* gene disruptions on growth of fish, we compared the standard length and body weight of mutants, including heterozygotes (*nt5ea*^+/∆2^ and *nt5eb*^+/∆10^) and homozygotes (*nt5ea*^∆2/∆2^ and *nt5eb*^∆10/∆10^), to those of wild-type fish at 12 weeks post-hatching. As shown in Table 1, the standard length of heterozygotes (*nt5ea*^+/∆2^: 27.8 ± 0.7 mm and *nt5eb*^+/∆10^: 27.2 ± 0.8 mm) and homozygotes (*nt5ea*^∆2/∆2^: 28.0 ± 0.9 mm and *nt5eb*^∆10/∆10^: 27.5 ± 0.7 mm) were not significantly different from that of the wild type (27.6 ± 1.0 mm). The body weight of heterozygotes (*nt5ea*^+/∆2^: 327.2 ± 17.1 mg and *nt5eb*^+/∆10^: 316.1 ± 20.0 mg) and homozygotes (*nt5ea*^∆2/∆2^: 338.2 ± 20.8 mg and *nt5eb*^∆10/∆10^: 321.6 ± 21.7 mg) were also not significantly different from that of wild-type fish (324.3 ± 19.1 mg). Subsequently, we dissected two male and two female wild-type, heterozygous (*nt5ea*^+/∆2^ and *nt5eb*^+/∆10^), homozygous (*nt5ea*^∆2/∆2^ and *nt5eb*^∆10/∆10^) fish and histologically analyzed gills and gallbladders in which *nt5ea* or *nt5eb* was highly expressed (Fig. S8a–f). The histological evaluation showed no remarkable differences in the appearance or structure of organs between wild type and *nt5e* mutants and no morphological alterations such as cell hypertrophy in *nt5e* mutants (Fig. 5a–f).

**Table 1:** Body size evaluation of wild-type and *nt5e* mutant medaka.

Genotype	Standard length (mm)				Body weight (mg)		
	Mean	SD	*P*		Mean	SD	*P*
wild type	27.6	1.0	–		324.3	19.1	–
*nt5ea*^∆2^ heterozygotes	27.8	0.7	NS		327.2	17.1	NS
*nt5ea*^∆2^ homozygotes	28.0	0.9	NS		338.2	20.8	NS
*nt5eb*^∆10^ heterozygotes	27.2	0.8	NS		316.1	20.0	NS
*nt5eb*^∆10^ homozygotes	27.5	0.7	NS		321.6	21.7	NS

The data shown were obtained with ten individual fish (n = 10 for each).

NS means not significantly different from wild type with one-way ANOVA followed by Tukey’s HSD test (*P* > 0.05).

## Discussion

In fish, IMP-degrading activities have been detected in the extracts from several tissues^24,25^; however, the genes responsible for the IMP degradation have not been elucidated. Thus, this is the first report of the successful identification of molecules that promote IMP degradation in teleosts. Unlike mammals, many teleost fish generally have more than one copy of a gene in their genomes due to whole genome duplication^33^. It has been reported that mammals, such as humans and mice, have only a single copy of *nt5e*^23^, whereas we found that medaka possessed two copies of *nt5e*, namely, *nt5ea* and *nt5eb*. RNA-seq analysis showed that *nt5ea* and *nt5eb* were highly expressed in the gills and gallbladder, respectively, and that their expression patterns were different. Furthermore, embryo-based expression analysis revealed that both *nt5ea* and *nt5eb* are involved in IMP metabolism. The different expression patterns and sharing of IMP degradation in *nt5ea* and *nt5eb* indicate that medaka *nt5e* underwent sub-functionalization after duplication. The nucleotide sequences of *nt5ea* and *n5eb* isolated from the Cab strain were slightly different from those from the Hd-rR strain. We speculate that the point mutations identified were due to differences between Cab and Hd-rR strains. In support of this notion, Morimoto et al.^34^ reported several silent and missense mutations in other common gene sequences between the Cab and Hd-rR strains.

We also found two differences between the knockout of *nt5ea* and *nt5eb*. First, the inheritance mode of the mutant trait was different in the *nt5e*a and *nt5eb* strains. The heterozygous *nt5ea* mutants had higher IMP levels than the wild type, showing a reduction in IMP-degrading activity. This result suggests that the heterozygous *nt5ea* mutants did not possess sufficient IMP-degrading activity to match that of wild-type *nt5ea*, indicating haploinsufficiency of *nt5ea*^35^. In contrast, the heterozygous *nt5eb* mutants exhibited temporal changes in IMP levels that were consistent with those in the wild type. We assumed that the heterozygous *nt5eb* mutants expressed sufficient amounts of functional Nt5eb from a single intact allele such that haploinsufficiency was not induced, unlike in the case of *nt5ea*. Because the reasons for these functional differences in alleles at the *nt5ea* and *nt5eb* loci are unknown, a more detailed functional analysis of each gene is required. The second difference is that the retention effect of IMP was higher when *nt5ea* was disrupted than when *nt5eb* was disrupted. The IMP contents of *nt5ea* homozygous mutant strains at 96 h postmortem (*nt5ea*^∆2/∆2^: 3.58 ± 0.25 and *nt5ea*^∆5,521/∆5,521^: 3.96 ± 0.36) were significantly higher than those of *nt5eb* homozygous mutant strains (*nt5eb*^∆10/∆10^: 2.88 ± 0.34 and *nt5eb*^∆10,900/∆10,900^: 2.63 ± 0.17). The IMP contents of *nt5ea* homozygous mutant strains at 48 h postmortem (*nt5ea*^∆2/∆2^: 3.67 ± 0.37 and *nt5ea*^∆5,521/∆5,521^: 4.02 ± 0.45) were also significantly higher than those of *nt5eb* homozygous mutant strains (*nt5eb*^∆10/∆10^: 3.13 ± 0.23 and *nt5eb*^∆10,900/∆10,900^: 3.04 ± 0.25), respectively. In contrast, our RNA-seq data showed that transcripts of *nt5ea* in skeletal muscle (male: 3.12 reads per kilobase of exon per million mapped sequence reads (RPKM) and female: 2.70 RPKM) were slightly more or equal to those of *nt5eb* (male: 2.22 RPKM and female: 1.70 RPKM). Additionally, our expression analysis using embryos showed that the IMP-degrading activity of Nt5ea was significantly higher than that of Nt5eb. Given that the transcript levels of *nt5ea* and *nt5eb* were almost equal and that Nt5ea had higher IMP-degrading activity than Nt5eb, we suggest that Nt5ea is the dominant enzyme responsible for IMP degradation in skeletal muscle, which may explain why the IMP levels in the *nt5ea* mutants were higher than those in the *nt5eb* mutants.

In contrast, each of the knockout strains that showed the retention effects of IMP had one common feature. In the measurement analysis from 0 to 24 h postmortem, there was no change in the IMP levels among all groups tested, whereas the IMP contents of mutants, including heterozygotes (*nt5ea*^+/–^) and homozygotes (*nt5ea*^–/–^ and *nt5eb*^–/–^), were significantly higher than that of the wild type after 48 h postmortem. The IMP retention effects via *nt5ea* or *nt5eb* disruption occurred 2 days after death, potentially due to both the collapse of the cell membrane and the in vivo localization of Nt5e. In general, cell membranes in meat gradually disintegrate during the ripening period, followed by the leakage of intracellular fluid^36^. Medaka Nt5ea and Nt5eb in muscle may be responsible for the degradation of intracellular IMP. Our in silico analysis using SignalP revealed N-terminal secretory signal peptides in Nt5ea and Nt5eb, suggesting that both enzymes were distributed extracellularly. Additionally, mammalian Nt5e has been reported to attach to the extracellular membrane by a glycosyl phosphatidylinositol anchor^37^. These facts suggest that IMP begins to degrade in the wild type after a delay of several days postmortem. Therefore, the effect of IMP maintenance by *nt5ea* and *nt5eb* disruption may also have appeared several days after death.

We utilized two different knockout approaches, including single sgRNA-directed mutagenesis and dual sgRNA-directed large gene deletion, to generate *nt5e*-mutants with small and large deletions, respectively. Although this strategy was practical enough to exploit for medaka, it may be difficult to apply to cultured fish. Farmed fish other than medaka often require considerable time and effort to establish the desired genome-engineered strain^38^. Therefore, a reasonable strategy for genetic disruption of cultured fish is to use mainly single sgRNA-directed mutagenesis and, if funds and facilities for breeding are available, to use dual sgRNA-directed large gene deletion as well. The degree of postmortem change in IMP of mutants with large and small deletions produced by the two knockout methods was fairly consistent. The results suggest that small deletions were sufficient to interfere with the functions of *nt5ea* and *nt5eb* genes. Because the edited fish with small deletions have been officially approved as food-grade in Japan^29^, our results using medaka provide a baseline example for future studies on other cultured fish as well.

This study provides other important insights into the potential application of this method to other fish species. Our knockout medaka appeared normal and had no obvious health, growth, or reproduction problems (up to the F_6_ generation). The body size and tissue structure of *nt5e*-mutants were comparable to those of the wild type. In addition, *nt5e*^–/–^ mice have been reported to have normal fertility and no developmental abnormalities^39^. Taking these and our results into consideration, it is suggested that the disruption of *nt5e* function, even in other fish, may not have unfavorable effects (e.g., embryonic lethality). Based on the results of our in silico analysis with other fish Nt5e sequences, *T. rubripes*, *O. niloticus*, and *S. dumerili*, which belong to the order of Perciformes, showed high similarities to Nt5ea and Nt5eb in medaka fish (Nt5ea: 78.8–81.3%, Nt5eb: 77.5–81.5%). Therefore, it is likely that the Nt5e function is more conserved in species belonging to Perciformes than in other taxonomic groups. Our research group has established new breeds in aquaculture marine fish such as *T. rubripes* and the perciform *P. major*, using the CRISPR-Cas9 system^27–29^. We are planning to evaluate whether the editing of cultured fish *nt5e* can lead to the production of meat with enhanced umami taste. The CRISPR-based method for IMP maintenance described here would be effective not only for marine products, but also for livestock products. A genome-wide association study (GWAS) revealed that two non-synonymous single nucleotide polymorphisms (SNPs) in bovine Nt5e affected the degradation rate of IMP by regulating Nt5e enzymatic activity^40^. Because practical genome editing methods using the CRISPR-Cas9 system have already been established in livestock, such as cattle and pigs^41,42^, *nt5e* mutants can be simply and rapidly produced. The functional disruption of *nt5e* may contribute to increased meat quality in a wide range of organisms.

In conclusion, we demonstrated that *nt5e* knockout was useful for postmortem maintenance of IMP levels in muscles of medaka. Because the breeding of aquatic products lags far behind that of agricultural and livestock products, there is still much room for genetic improvement of farmed fish. Further research into the IMP-degradation system will provide a platform for molecular breeding using genome editing technology, leading to establishment of new sources of meat with superior umami taste.

## Materials and methods

### Fish

The Cab closed colonies were kindly gifted by the group of Dr. Todo at Radiation Biology Center, Kyoto University, Kyoto, Japan. Fish were maintained in an aquarium at 26 °C under a 14/10-h light/dark cycle. All animal experiments were conducted according to the relevant national (Act on Welfare and Management of Animals, Ministry of the Environment, Japan) and international guidelines. All animal experiments were approved by the Animal Experimentation Committee of Kyoto University (No. 31-45) and of Kindai University (KAAG-2021-014). Our research was performed in accordance with the ARRIVE guidelines.

### Identification and cloning of nt5ea and nt5eb in medaka

The NCBI genome database of the medaka Hd-rR strain (GenBank accession number ASM223467v1) was screened with the zebrafish *nt5e* nucleotide sequence (GenBank accession number 393906) as a query. The transcription levels of *nt5e* genes were examined using our previous RNA-seq data (DDBJ accession number DRA014727)^43^. This expression profile was constructed using the total RNA of 10 different tissues from the Cab males and females, which helped select the tissues and sex for subsequent RNA extraction. The CDSs of the Cab Nt5ea and Nt5eb were isolated by RT-PCR as follows. Total RNA was extracted from the male gills or gallbladder of the Cab strain using an RNeasy Plus Mini kit (Qiagen, Hilden, Germany). One microgram of total RNA was used to synthesize first-strand cDNA with random hexamers and SuperScript III Reverse Transcriptase (Thermo Fisher Scientific, Waltham, MA, USA), according to the manufacturer’s instructions. The cDNA was used for PCR amplification of the coding sequences of *nt5ea* or *nt5eb* with KOD-FX DNA polymerase (TOYOBO, Osaka, Japan) using the primers nt5ea-RT-FW/nt5ea-RT-FW or nt5eb-RT-FW/nt5eb-RT-FW (Table S3). The PCR conditions were as follows: incubation at 94 °C for 2 min, followed by 35 cycles at 98 °C for 10 s, 58 °C for 30 s, and 68 °C for 50 s. The PCR products in the agarose gels were purified using a NucleoSpin Gel and PCR Clean-up kit (Macherey-Nagel, Düren, Germany). The amplicons of *nt5ea* and *nt5eb* were cloned to construct the plasmids pCS2_nt5ea_LUC_EGFP and pCS2_nt5eb_LUC_EGFP (Fig. S1b) using In-Fusion^®^ HD Cloning Kit (Takara, Shiga, Japan) following manufacturer instructions. To evaluate the IMP-degrading activities of Nt5ea and Nt5eb, pCS2_LUC_EGFP was used as the control plasmid. The construction of these plasmids is described in Supplementary Fig. 1a,b. All plasmids in this study were extracted using the Wizard^®^ Plus SV Minipreps DNA Purification System Kit (Promega, Madison, WI, USA) following manufacturer instructions and were sequenced using the Sanger method (data not shown).

### In silico analysis of Nt5ea and Nt5eb

Alignment of the AA and nucleotide sequences was performed using Clustal Omega (http://www.ebi.ac.uk/Tools/msa/clustalo/). Signal peptide cleavage sites were predicted using SignalP 6.0 Server (https://services.healthtech.dtu.dk/service.php?SignalP-6.0). CDSs of *nt5e* across teleost and non-teleost vertebrates (*Homo sapiens*, *Bos taurus*, and *Gallus gallus*) were collected using ORTHOSCOPE v1.5.1 (http://yurai.aori.u-tokyo.ac.jp/orthoscope/Vertebrata.html) and BLAST search (https://blast.ncbi.nlm.nih.gov/Blast.cgi). Percentage AA sequence identities and similarities were calculated using Ident and Sim Analysis (https://www.bioinformatics.org/sms2/ident_sim.html). Phylogenetic analysis was performed using the neighbor-joining (NJ) method in the MEGA X program and bootstrap sampling was repeated 1,000 times.

### Overexpression of Nt5ea and Nt5eb through microinjection of RNA

Three RNAs encoding *LUC* and *EGFP* were synthesized and purified as previously described^43^. Briefly, pCS2 plasmids, pCS2-LUC-EGFP-pA, pCS2-nt5ea-LUC-EGFP-pA, and pCS2-nt5eb-LUC-EGFP-pA (Fig. S1a,b), were linearized with NotI and purified using the NucleoSpin Gel and PCR Clean-up kit (Macherey-Nagel) for in vitro transcription. The capped RNA was transcribed from the purified DNA templates using the Message mMachine SP6 Kit (Life Technologies, Carlsbad, CA, USA). The synthesized RNA was purified using an RNeasy Mini Kit (Qiagen) for microinjection. Approximately 2–4 nL of 100 ng/µL of each RNA was injected into the cytosol of embryos at the one-cell stage, as previously described^44^. The injected embryos were observed under an SZX16 fluorescence stereomicroscope with the SZX2-FGFP filter set (Olympus, Tokyo, Japan). Microscopic images were captured using a DP73 camera and cellSens image acquisition software (Olympus).

### Assay of IMP-degrading activity in Nt5ea and Nt5eb

Forty embryos with green fluorescence at 1 dpf were pooled in a 1.5-mL microtube containing 50 μL of phosphate-buffered saline (PBS) (pH 7.4) and then homogenized with a pestle. The same number of embryos without microinjection was used as the control. Each homogenate was centrifuged at 15,000×*g* for 10 min at 4 °C and the pellet was discarded. The supernatant was added to 150 µL of PBS, and the resultant solution was separately used for the reporter assay of LUC and the measurement of IMP degradation activity. The reporter assay was performed to normalize the expression amount of each RNA injected, because translation efficiency generally varies by RNA length and codon^45^. Then, 25 µL of each supernatant diluted with PBS was frozen (− 80 °C) and thawed at approximately 20 °C to ensure lysis of cell membranes, and then mixed with 75 µL of the Reporter Lysis Buffer (Promega). One hundred microliters of each lysate were added to a well of a 96-well microplate, and the luminescent reaction was initiated by adding 10 μL of prepared luciferase assay reagent II, including the substrate with assay buffer, provided in the DLR assay system (Promega) to each well. Bioluminescence intensity was measured using a GloMax 96 microplate luminometer (Promega). Next, to validate the IMP degradation activities of Nt5ea and Nt5eb, 25 µL of each supernatant diluted with PBS was added to the reaction mixture containing 50 mM Tris–HCl (pH7.2) and 5 mM MgCl_2_ in a final volume of 50 µL. After preincubation of each mixture for 10 min at 28 °C, the reaction was started by adding the substrate IMP to a final concentration of 100 µM, and was stopped by adding 100 µL of 10% perchloric acid. Each sample was neutralized with 1 N KOH on ice and centrifuged at 15,000 g for 10 min at 4 °C. The supernatant was filtered through a 0.45 µm filter (Merck Millipore, Burlington, MA, USA), and IMP was measured by HPLC using a column (TSK gel ODS 80Ts, 4.6 × 250 mm; Tosoh, Tokyo, Japan). Chromatographic separation was obtained by increasing the acetonitrile concentration in the mobile phase of 0.1 M NaH_2_PO_4_ (pH 4.1)^46^.

To compare the IMP-degrading activities of Nt5ea and Nt5eb, the expression level of the LUC in the Nt5ea group was set to “1” and the relative to that in the Nt5eb group was calculated. This relative value was used to divide the amount of IMP degradation between each time point in the Nt5eb group, thereby normalizing the difference in expression levels between the Nt5ea and Nt5eb groups.

### Preparation of sgRNAs for microinjection

All sgRNAs for *nt5ea* and *nt5eb* were synthesized via a cloning-free method. The template DNA for the sgRNAs was amplified by PCR using KOD-FX DNA polymerase (TOYOBO) and three oligonucleotides: each OligoA (Table S3), OligoB, and OligoC^43^. The PCR program was as follows: one cycle at 94 °C for 2 min followed by 35 PCR cycles of 98 °C for 10 s, 58 °C for 30 s and 68 °C for 5 s. The resulting PCR products were purified using the NucleoSpin Gel and PCR Clean-up Kit (Macherey-Nagel) and then transcribed in vitro using the CUGA^®^7 gRNA Synthesis Kit (Nippon Gene, Tokyo, Japan). All synthesized sgRNAs were purified using an RNeasy Mini Kit (Qiagen) for microinjection.

### Microinjection of CRISPR-Cas9 components for nt5ea or nt5eb knockout

To evaluate the DSB-inducing activity of sgRNAs, an injection mixture containing 100 ng/µL of each sgRNA including sgRNA-nt5ea-1, sgRNA-nt5ea-2, sgRNA-nt5eb-1, or sgRNA-nt5eb-2, and 500 ng/µL of Cas9 protein (Integrated DNA Technology) was prepared. Single sgRNA-directed mutagenesis was performed by preparing an injection mixture containing 100 ng/µL of sgRNA-nt5ea-1 or sgRNA-nt5eb-1 and 500 ng/µL Cas9 protein (Integrated DNA Technology) was prepared. To perform dual sgRNA-directed large gene deletion, an injection mixture containing 100 ng/µL of each pair of sgRNAs, including sgRNA-nt5ea-1/sgRNA-nt5ea-2 for *nt5ea* or sgRNA-nt5eb-1/sgRNA-nt5eb-2 for *nt5eb*, and 500 ng/µL of Cas9 protein (Integrated DNA Technology) was prepared. Approximately 2–4 nL of each mixture was injected into one-cell-stage embryos^44^.

### Establishment of knockout strains with frame-shifts or large deletions

A PCR-based analysis was performed to detect genetic mutations induced by CRISPR components. Genomic DNA was extracted from whole embryos or caudal fins of adult fish using the alkaline lysis buffer method^44^. The DNA fragment (200–500 bp) containing *nt5ea* or *nt5eb* was amplified by PCR with KOD-FX (TOYOBO) and each primer set (Fig. S6a, c, e, g, Table S3). The PCR conditions were as follows: incubation at 94 °C for 2 min, 35 cycles at 98 °C for 10 s, 58 °C for 30 s, and 68 °C for 5 s (amplicons < 500 bp) or 15 s (amplicons = 500 bp). The resulting amplicons were analyzed using a microchip electrophoresis system with MCE-202 MultiNA and DNA-500 Reagent Kit (Shimadzu, Kyoto, Japan) for HMA analysis^47^ or 1% agarose gel electrophoresis and were then directly sequenced using the Sanger method (data not shown).

### Quantification of ATP-related compounds in muscle

Each fish was anesthetized with 0.03% tricaine methanesulfonate (MS-222) at 12 weeks post-hatching; heads and internal organs were removed using fine forceps and scissors under a stereomicroscope. The dressed fish were stored in a plastic container at 4 °C. A Petri dish filled with water was also placed inside the container to prevent the meat surface from drying. At intervals, the fish was taken from storage at 4 °C, and a portion (~ 10.0 mg) of the meat was homogenized with 10% perchloric acid. The homogenate was centrifuged at 15,000×*g* for 10 min at 4 °C. The supernatant was neutralized with 1 N KOH on ice and centrifuged at 15,000×*g* for 10 min at 4 °C. The supernatant was filtered through a 0.45 µm filter (Merck Millipore), and components were separated by HPLC using a column (TSK gel ODS 80Ts, 4.6 × 250 mm; Tosoh) and evaluated. Separation was achieved by increasing the acetonitrile concentration in 0.1 M NaH_2_PO_4_ (pH 4.1)^46^. The *K*-value was calculated as the percentage of HxR and Hx to the sum of ATP and degradation products, as follows^48^:

$$\mathrm{K}-\mathrm{value }(\mathrm{\%}) = (\mathrm{HxR }+\mathrm{ Hx})/(\mathrm{ATP }+\mathrm{ ADP }+\mathrm{AMP }+\mathrm{ IMP }+\mathrm{ HxR }+\mathrm{ Hx}) \times 100$$

### Body measurements and histological staining

Adult medaka were anesthetized using MS-222 at 12 weeks post-hatching and standard length and body weight were measured. Gills and gallbladders were removed and fixed in Bouin’s solution for 20 h, and then stored in 70% ethanol at 4 °C until needed. For histological analysis, the fixed organs were embedded in paraffin, cut into 6-µm-thick cross sections, and stained with hematoxylin and eosin. The sections were visualized using an Eclipse E600 microscope (Nikon, Tokyo, Japan). Images were acquired with a DP73 camera and cellSens image acquisition software (Olympus).

### Statistical analysis

All statistical analyses were performed using the R software (http://www.r-project.org). IMP degrading activity, IMP content of fish meat, *K*-value, standard length, and body weight between groups were compared using one-way ANOVA followed by Tukey’s HSD test. LUC luminescence was analyzed using Student’s *t*-test. *P* < 0.05 was considered significant.

## Supplementary Information

Supplementary Legends.

Supplementary Figure 1.

Supplementary Figure 2.

Supplementary Figure 3.

Supplementary Figure 4.

Supplementary Figure 5.

Supplementary Figure 6.

Supplementary Figure 7.

Supplementary Figure 8.

Supplementary Table 1.

Supplementary Table 2.

Supplementary Table 3.

## Data Availability

The sequences of Cab *nt5ea* and *nt5eb* were deposited in the database (*nt5ea*: Genbank accession number ON873732 and *nt5eb*: Genbank accession number ON873733).
