# Supplementary material for: Targeted deletion of ecto-5′-nucleotidase results in retention of inosine monophosphate content in postmortem muscle of medaka (Oryzias latipes)
Source: Sci Rep. 2022 Nov 3;12:18588. doi: 10.1038/s41598-022-22029-y (PMC9633828; doi:10.1038/s41598-022-22029-y)
Supplement: Supplementary file 1 — Supplementary Legends. [file 41598_2022_22029_MOESM1_ESM.docx]

***Scientific reports***

**Title**

Targeted deletion of *ecto-5′-nucleotidase* results in retention of inosine monophosphate content in postmortem muscle of medaka (*Oryzias latipes*)

**Authors**

Yu Murakami^1*^, Masashi Ando^1^, Ryota Futamata^2^, Tomohisa Horibe^3^, Kazumitsu Ueda^4^, Masato Kinoshita^5^ and Toru Kobayashi^1^

**Author Affiliations**

^1^Department of Fisheries, Graduate School of Agriculture, Kindai University, Nakamachi 3327-204, Nara 631-8505, Japan

^2^Division of Applied Life Sciences, Graduate School of Agriculture, Kyoto University, Kitashirakawa-Oiwake-cho, Sakyo-ku, Kyoto 606-8502, Japan

^3^Department of Medical-Bioscience, Faculty of Bio-Science, Nagahama Institute of Bio-Science and Technology, 1266 Tamura-cho Nagahama-shi, Shiga 526-0829, Japan

^4^Institute for Integrated Cell-Material Sciences (WPI-iCeMS), KUIAS, Kyoto University, Kyoto 606-8501, Japan

^5^Division of Applied Biosciences, Graduate School of Agriculture, Kyoto University, Kitashirakawa-Oiwake-cho, Sakyo-ku, Kyoto 606-8502, Japan

*Corresponding author

Postal address: Kindai University, Nakamachi 3327-204, Nara 631-8505, Japan.

E-mail: ns_you-0347@nara.kindai.ac.jp; Tel: +81-742-43-6149; Fax: +81-742-43-1316

**Supplementary Information**

Additional Supporting Information can be found online in the Supplementary Information tab for this article:

**Fig. S1.** Three plasmids used in this study. (**a**) Scheme for the construction of plasmid, pCS2_LUC_EGFP. A backbone fragment (backbone-A) containing an SP6 promoter, a ColE1 replication origin, and an ampicillin resistance gene, was amplified from the plasmid pCS2+hSpCas9 (Addgene #51815) using a primer pair (Backbone1-FW/Backbone1-RV) (Table S3). An *LUC-GFP* cassette containing firefly *luciferase* (*LUC*), *enhanced green fluorescent protein* (*EGFP*), and *pA* was amplified from the plasmid pDs-ChgH-LUC-EGFP^[1]^ via PCR using the primers LUC-GFP-FW/LUC-GFP-RV^[2]^. The *LUC-GFP* cassette shared 15-base homologous sequences (HS) with each end of a backbone-A. These two fragments were joined through In-Fusion reaction to construct pCS2_LUC_EGFP. (**b**) Scheme for the construction of the plasmids pCS2_nt5ea_LUC_EGFP and pCS2_nt5eb_LUC_EGFP. A backbone fragment (backbone-B) containing an SP6 promoter, a ColE1 replication origin, an ampicillin resistance gene, and the *LUC-GFP* cassette was amplified from the plasmid pCS2_LUC_EGFP using a primer pair (Backbone2-FW/Backbone1-RV) (Table S3). An insert fragment containing the coding sequence of *nt5ea* or *nt5e* shared 15-base homologous sequences (HS) with each end of a backbone-B. These two fragments were joined through In-Fusion reaction to construct pCS2_nt5ea_LUC_EGFP and pCS2_nt5eb_LUC_EGFP, respectively. A linker sequence (GGSGGT) was located between each *nt5e* and *LUC* or *LUC* and *EGFP* to increase the flexibility of the fusion protein consisting of Nt5ea or Nt5eb, LUC, and EGFP.

References

1. Murakami, Y., Horibe, T. & Kinoshita, M. Development of an efficient bioreactor system for delivering foreign proteins secreted from liver into eggs with a vitellogenin signal in medaka *Oryzias latipes*. *Fish. Sci.* **85**, 677–685 <https://doi.org/10.1007/s12562-019-01320-4> (2019).
2. Murakami, Y. & Kobayashi, T. An effective double gene knock-in strategy using small-molecule L755507 in the medaka fish (*Oryzias latipes*). *Genesis* **60**, e23465 <https://doi.org/10.1002/dvg.23465> (2022).

**Fig. S2.** Alignments of coding sequences of Cab and Hd-rR strains *nt5ea* (**a**) and *nt5eb* (**b**), respectively. The mutated region was extracted from the full-length sequence of *nt5ea* and *nt5eb*. Asterisks under each pair of aligned nucleotides indicate identity, whereas black boxes and white text show mismatch. Silent mutations are represented by a pink line, whereas missense mutation is surrounded by a green line. Nucleotide positions are indicated by the numbers on the left and right.

**Fig. S3.** *In silico* prediction of signal peptides in medaka Nt5ea and Nt5eb using SignalP. The predicted cleavage site (CS) in Nt5ea and Nt5eb is between position 28 and 29 (**a**) and 31 and 32 (**b**), respectively, as indicated by dark green dotted lines. SignalP predict secretory signal peptides that are transported through the Sec translocon and cleaved by Signal Peptidase I (Sec/SPI). The probabilities of the n-terminal (Sec/SPI n), center hydrophobic (Sec/SPI h), and c-terminal region (Sec/SPI c) of the signal peptides are indicted by red, orange, and yellow lines, respectively, whereas those of regions that are not signal peptides (OTHER) are indicated by pink dotted lines.

**Fig. S4.** Detection of LUC activity in embryos injected with RNA encoding *nt5ea*-*LUC*-*GFP* or *nt5eb*-*LUC*-*GFP*. The luciferase activity in the embryos was expressed as the fold change compared with the group Nt5ea_LUC_GFP that was arbitrarily set to “1”. Data are presented as the mean ± SD of single determination from triple experiments (n = 40/group). Asterisks (*) indicate that the values were significantly different between two groups by Student’s *t*-test (*p* < 0.05).

**Fig. S5.** Evaluation of the genomic mutations using HMA analysis. (**a**) The double strand break (DSB)-inducing activities of sgRNAs targeting to *nt5ea*. The target region was amplified via PCR with primer set nt5ea-HMA1-FW/nt5ea-HMA1-RV or nt5ea-HMA2-FW/nt5ea-HMA2-RV (Table S3). The electrophoretic image showed the three representative results of embryos injected with Cas9 and sgRNA-nt5ea-1 and those of embryos injected with Cas9 and sgRNA-nt5ea-2. (**b**) The DSB-inducing activities of sgRNAs targeting to *nt5eb*. The target region was amplified via PCR with primer set nt5eb-HMA1-FW/nt5eb-HMA1-RV or nt5eb-HMA2-FW/nt5eb-HMA2-RV (Table S3). The electrophoretic image showed the three representative results of embryos injected with Cas9 and sgRNA-nt5eb-1 and those of embryos injected with Cas9 and sgRNA-nt5eb-2. The control (Ctrl) showed a result from an embryo without injection (**a, b**).

**Fig. S6.** Establishment of *nt5ea*- or *nt5eb*-deficient strains. (**a, e**) Design of sgRNAs and primers for targeted mutagenesis at *nt5ea* or *nt5eb* locus. Blue and magenta letters show the target sequence and protospacer adjacent motif (PAM), respectively. Orange triangles indicate the cleavage sites recognized by Cas9 nuclease. 1F/1R and 4F/4R represent the PCR primer pairs used for mutation detection (nt5ea-HMA1-FW/nt5ea-HMA1-RV and nt5eb-HMA1-FW/nt5eb-HMA1-RV). Black and white boxes showed coding sequences (CDS) and untranslated regions (UTR), respectively. (**b, f**) Genotyping of mutants by using a heteroduplex mobility assay (HMA). Upper panels show electrophoretic images of the first and second HMA. Heterozygote exhibited a heteroduplex band pattern in the first HMA, as indicated by lanes with light blue letters. Homozygote showed a heteroduplex band pattern in the second HMA, as indicated by lanes with red letters. Cab embryos harboring no mutation were used as a control (Ctrl). Lower panels show the nucleotide sequences of wild type (WT) and *nt5ea*- or *nt5eb*-deficient medaka. (**c, g**) Design of sgRNAs and primers for large deletion at the *nt5ea* or *nt5eb* locus. Each pair of sgRNAs (sgRNA-nt5ea-1/sgRNA-nt5ea-2 or sgRNA-nt5eb-1/sgRNA-nt5eb-2) was co-injected with Cas9 protein into embryos. The first round of PCR was performed using the primer pair 2F/2R or 5F/5R (nt5ea-Large1-FW/nt5ea-Large1-RV or nt5eb-Large1-FW/nt5eb-Large1-RV), and the resultant amplicons from the intact alleles were 500 bp each. The second round of PCR was performed using the primer pair 3F/3R or 6F/6R (nt5ea-Large2-FW/nt5ea-Large2-RV or nt5eb-HMA1-FW/nt5eb-HMA2-RV), and the resultant amplicons from the mutated alleles were 325 or 328 bp in length, respectively. (**d, h**) Genotyping of mutants using a two-stage PCR analysis. Upper panels show electrophoretic images of the first and second PCR analysis. Heterozygotes exhibited a single band in both the first and second PCR analyses, as indicated by lanes with light blue letters. Homozygotes showed a single band in the second PCR analysis only, as indicated by lanes with red letters. Cab embryos harboring no mutation were used as a control (Ctrl). Lower panels show the nucleotide sequences of the wild type (WT) and *nt5ea*- or *nt5eb*-deficient medaka, respectively.

**Fig. S7.** Alteration of amino acid (AA) sequence of knockout fish with frame-shift mutations. (**a**) Alignment of predicted AAs between the *nt5ea* wild-type fish (*nt5ea*^wt/wt^) and the homozygous knock-out fish (*nt5ea*^∆2/∆2^). The deletion (*nt5ea*^∆2^) generates a truncated protein of 110 AA while the wild-type allele (*nt5ea*^wt^) generates an intact protein of 576 AA. (**b**) Alignment of predicted AAs between the *nt5eb* wild-type fish (*nt5eb*^wt/wt^) and the homozygous knock-out fish (*nt5eb*^∆10/∆10^). The deletion (*nt5eb*^∆10^) will generate a truncated protein of 105 AAs while the wild-type allele (*nt5eb*^wt^) generates an intact protein of 580 AAs. (**a, b**) Red asterisks indicate stop codons while black asterisks under each pair of AAs indicate identity. Black boxes and white text show mutated sequences.

**Fig. S8.** Histological observation of adult *nt5e* mutants. Gill of wild-type (**a**), *nt5ea*^+/∆2^ heterozygous (**b**), and *nt5ea*^∆2/∆2^ homozygous (**c**) fish at 12 weeks post-hatching. Gallbladder of wild-type (**d**), *nt5eb*^+/∆10^ heterozygous (**e**), and *nt5eb*^∆10/∆10^ homozygous (**f**) fish at 12 weeks post-hatching. Scale bars indicate 200 µM (**a, b, c**) and 150 µM (**d, e, f**), respectively.

**Table S1.** Nt5ea and Nt5eb amino acid sequence identity and similarity across various species.

**Table S2.** Results of microinjection and subsequent screening of founders.

**Table S3.** Oligonucleotide sequences of the primers used in this study.
