# Supplementary figures and images for: Targeted deletion of ecto-5′-nucleotidase results in retention of inosine monophosphate content in postmortem muscle of medaka (Oryzias latipes)
Source: Sci Rep. 2022 Nov 3;12:18588. doi: 10.1038/s41598-022-22029-y (PMC9633828; doi:10.1038/s41598-022-22029-y)

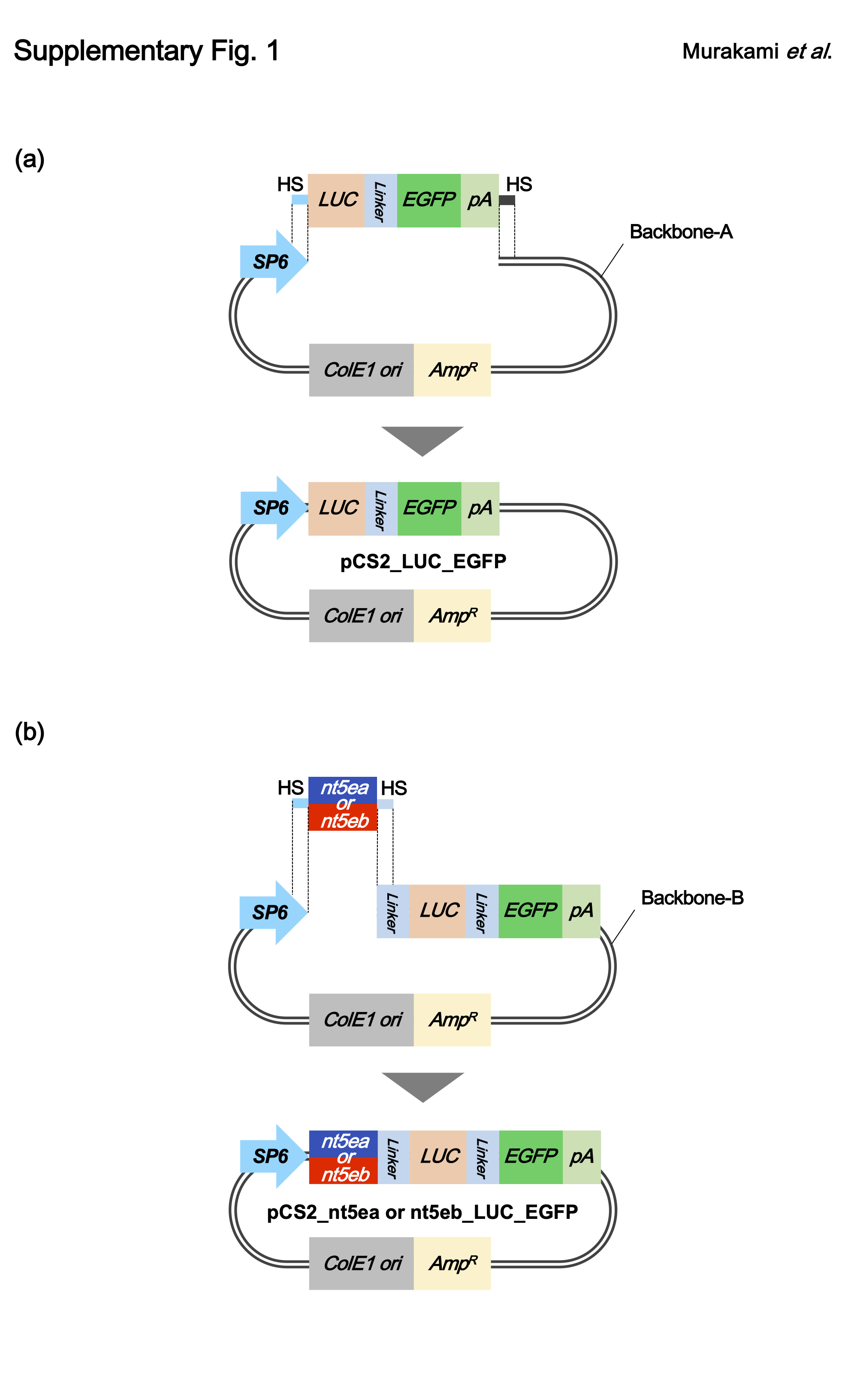

Supplement: Supplementary file 2 — Supplementary Figure 1. [file 41598_2022_22029_MOESM2_ESM.tiff]

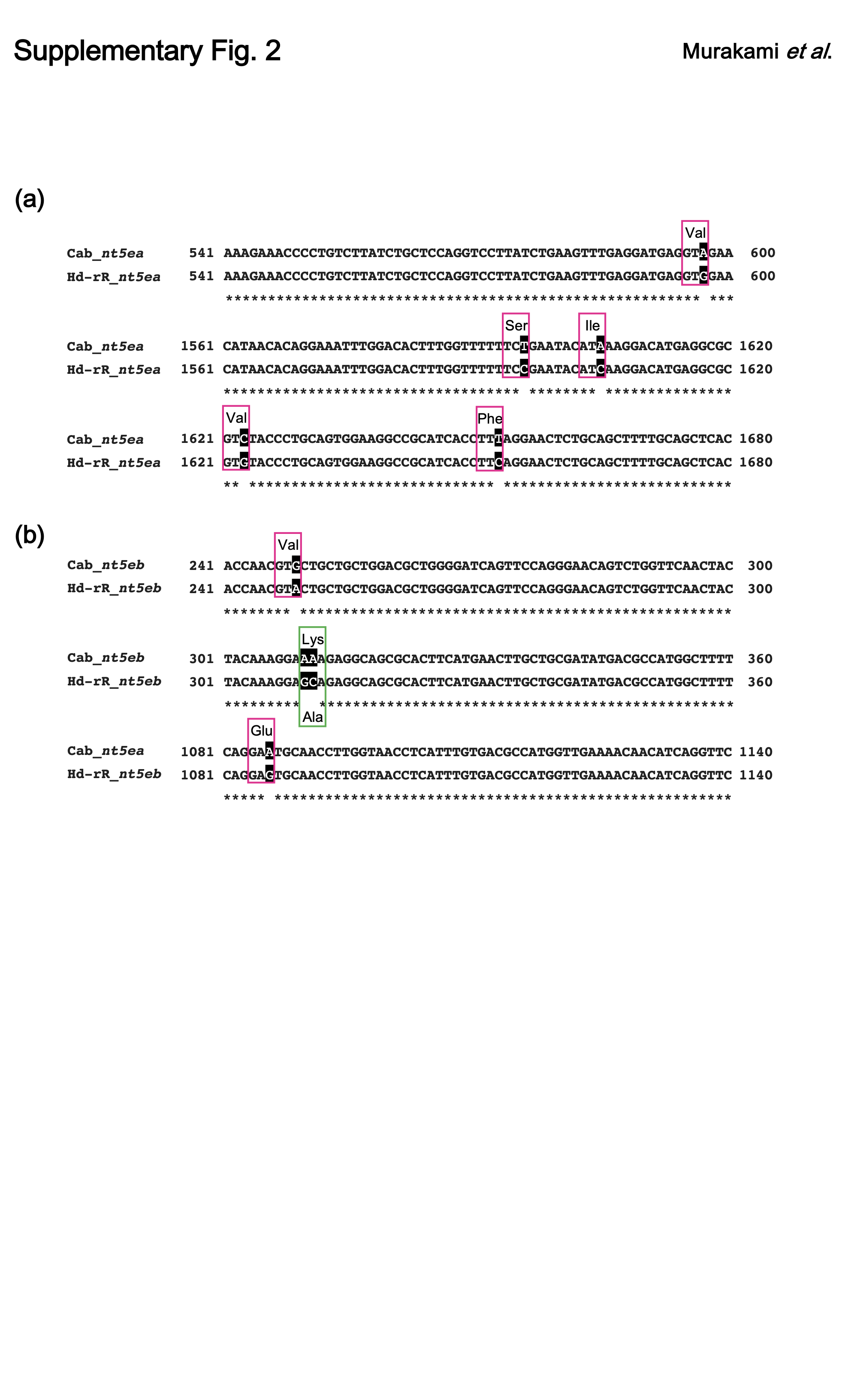

Supplement: Supplementary file 3 — Supplementary Figure 2. [file 41598_2022_22029_MOESM3_ESM.tiff]

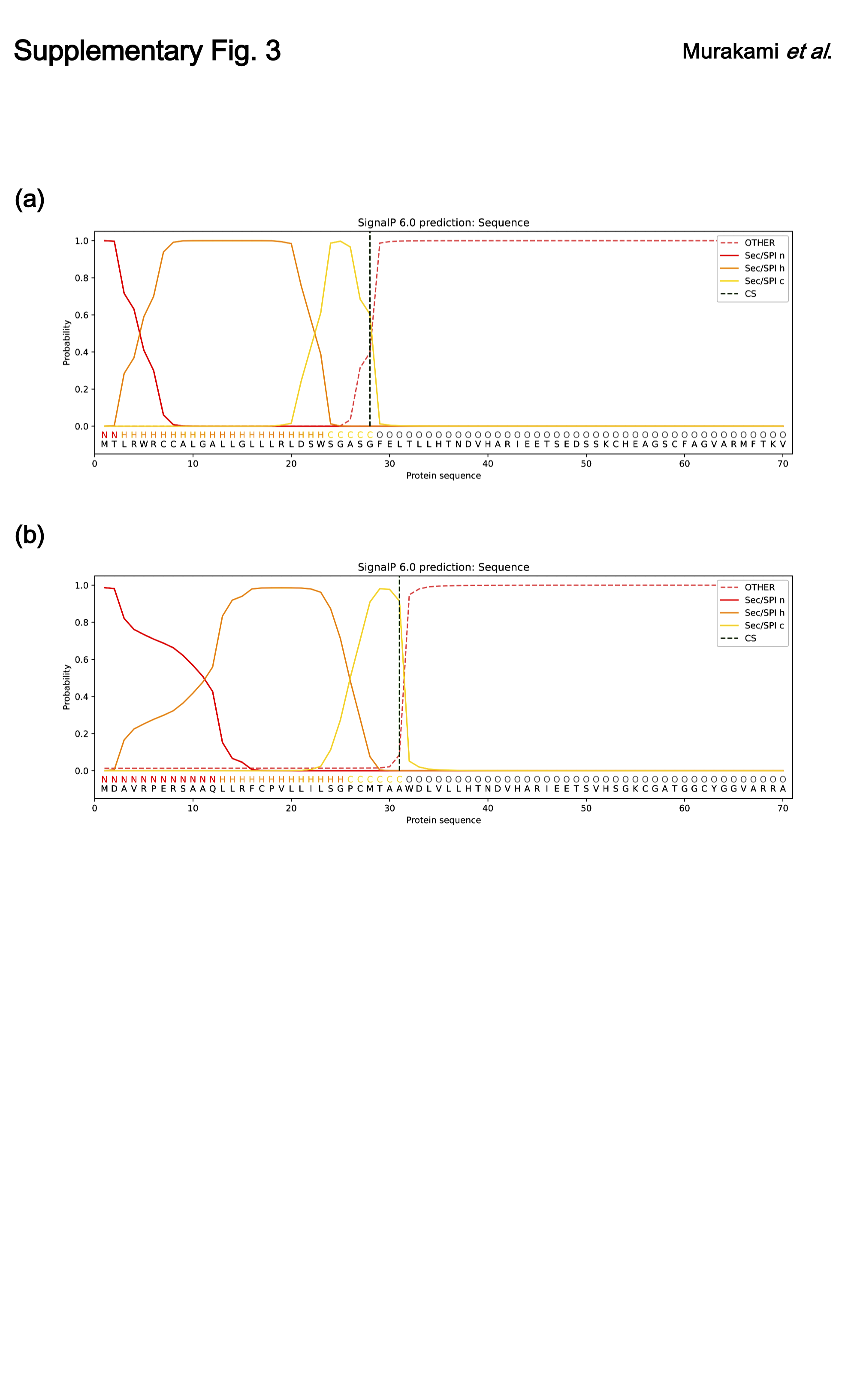

Supplement: Supplementary file 4 — Supplementary Figure 3. [file 41598_2022_22029_MOESM4_ESM.tiff]

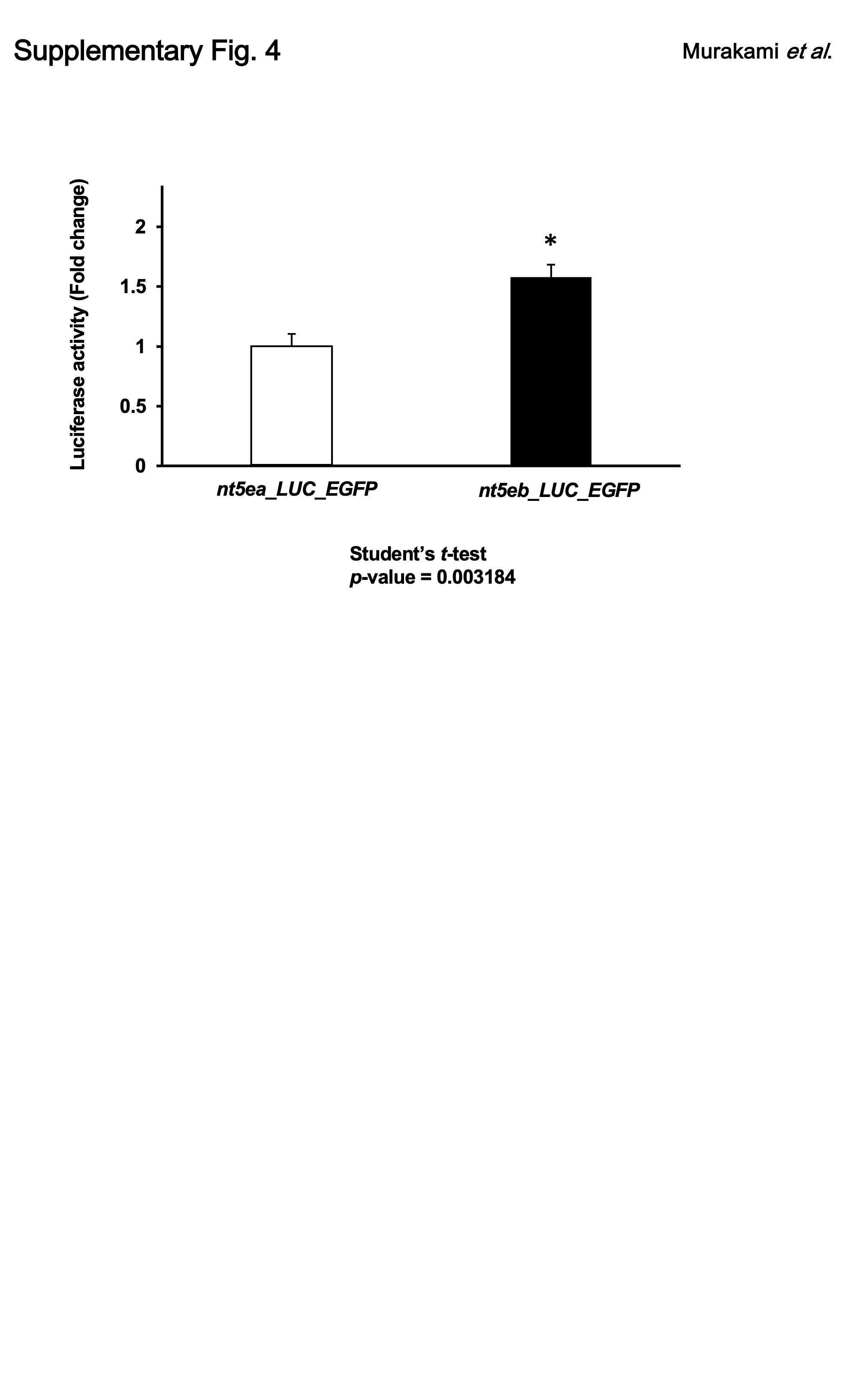

Supplement: Supplementary file 5 — Supplementary Figure 4. [file 41598_2022_22029_MOESM5_ESM.tiff]

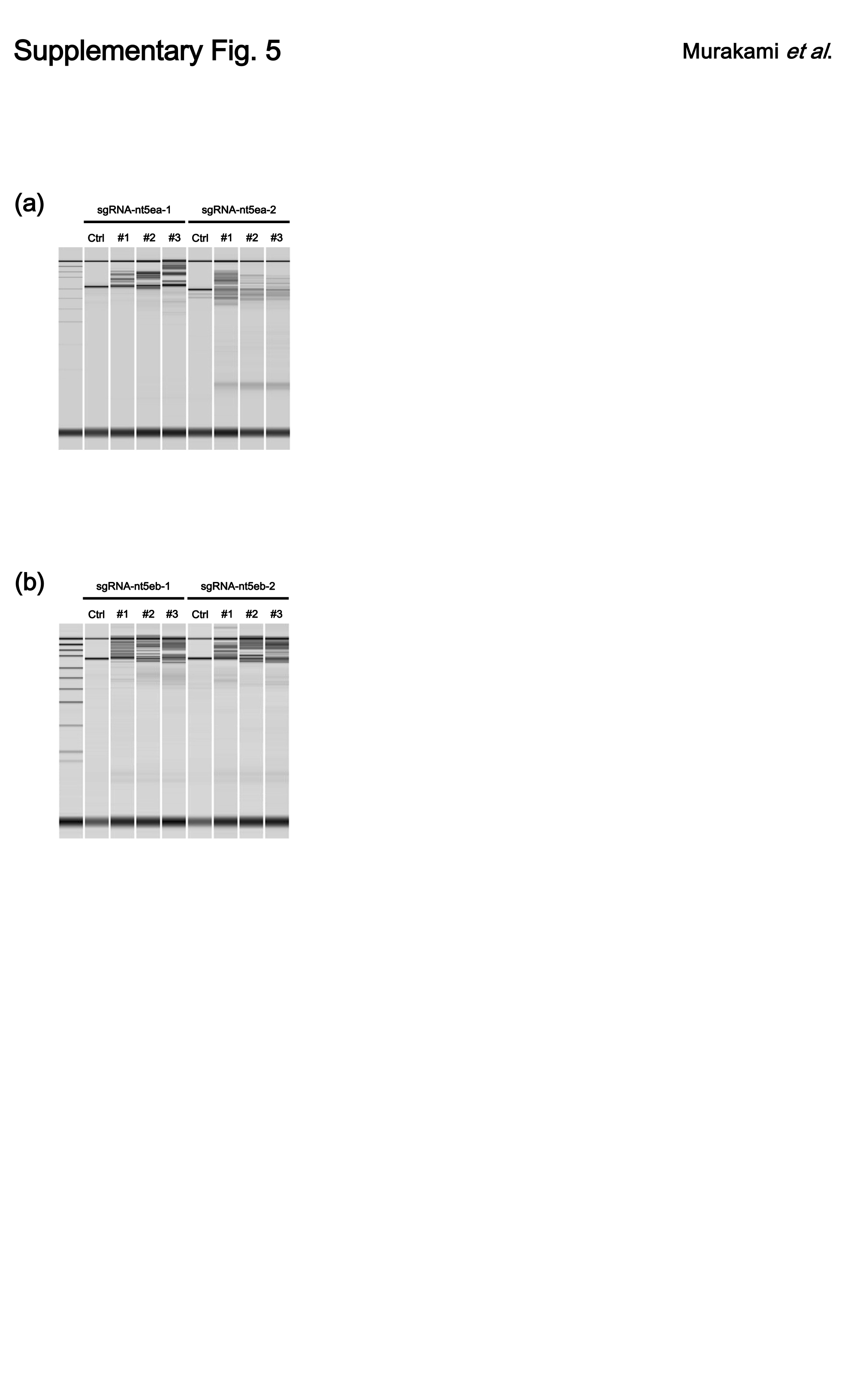

Supplement: Supplementary file 6 — Supplementary Figure 5. [file 41598_2022_22029_MOESM6_ESM.tiff]

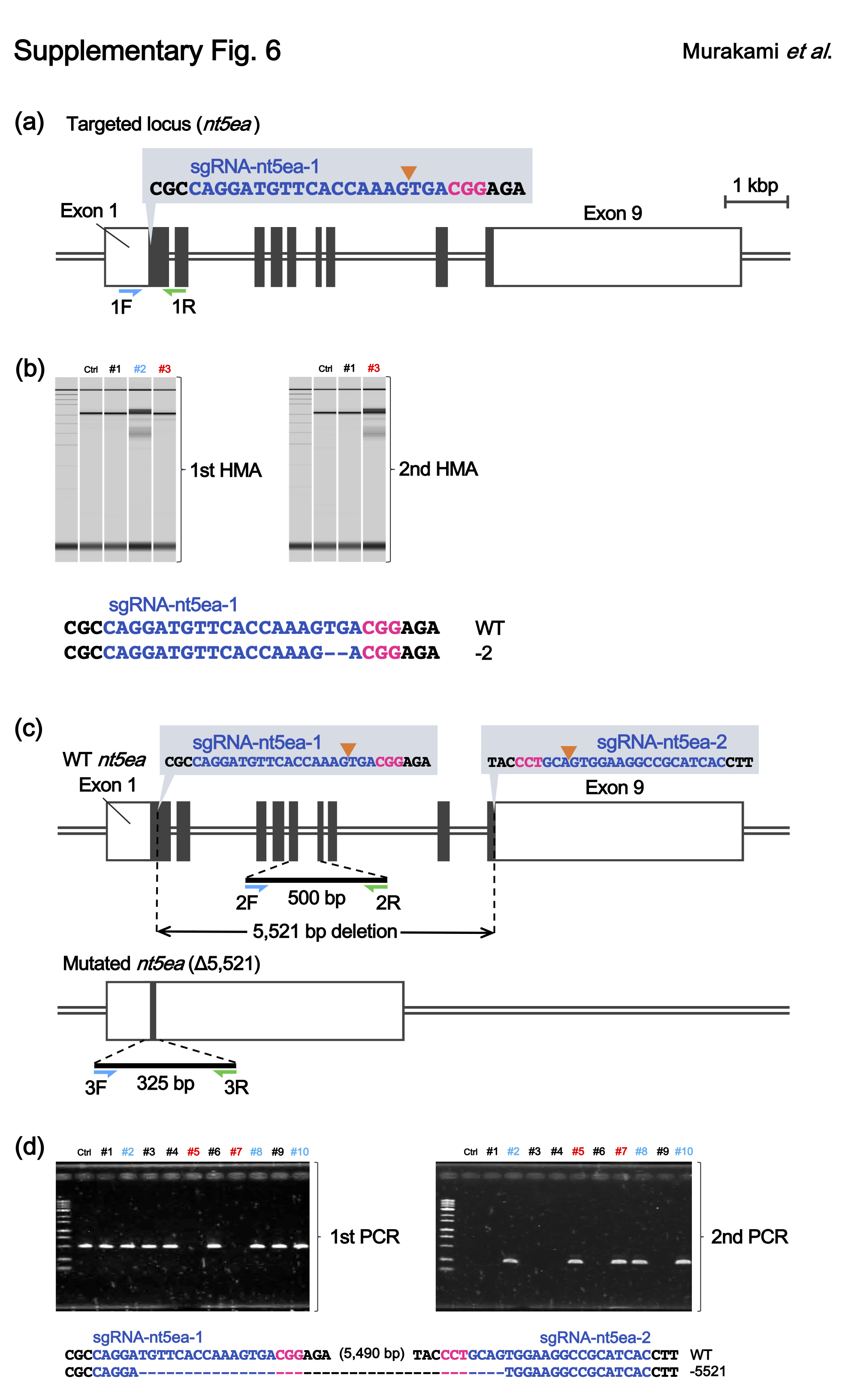

Supplement: Supplementary file 7 — Supplementary Figure 6. [file 41598_2022_22029_MOESM7_ESM.tiff]

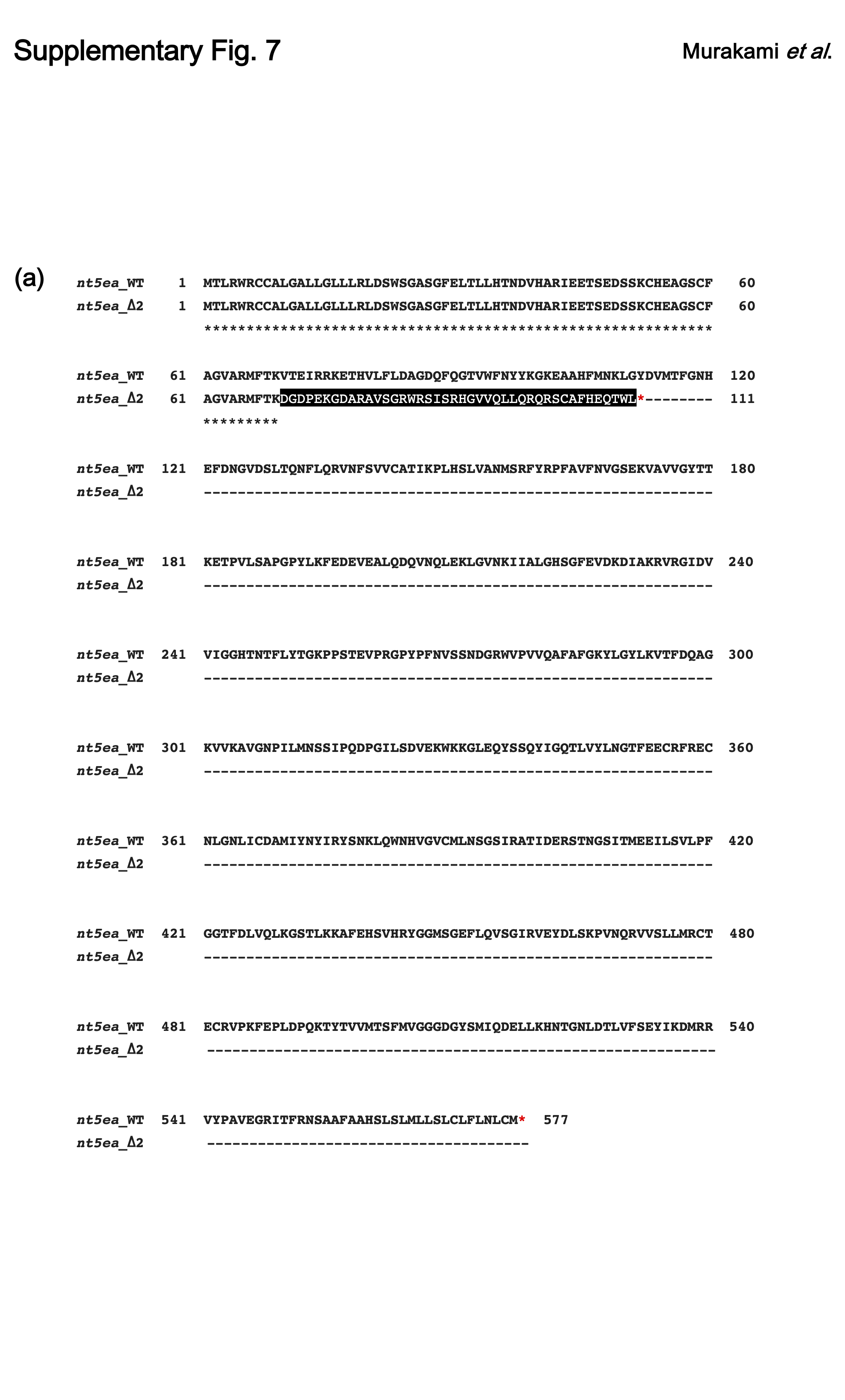

Supplement: Supplementary file 8 — Supplementary Figure 7. [file 41598_2022_22029_MOESM8_ESM.tiff]

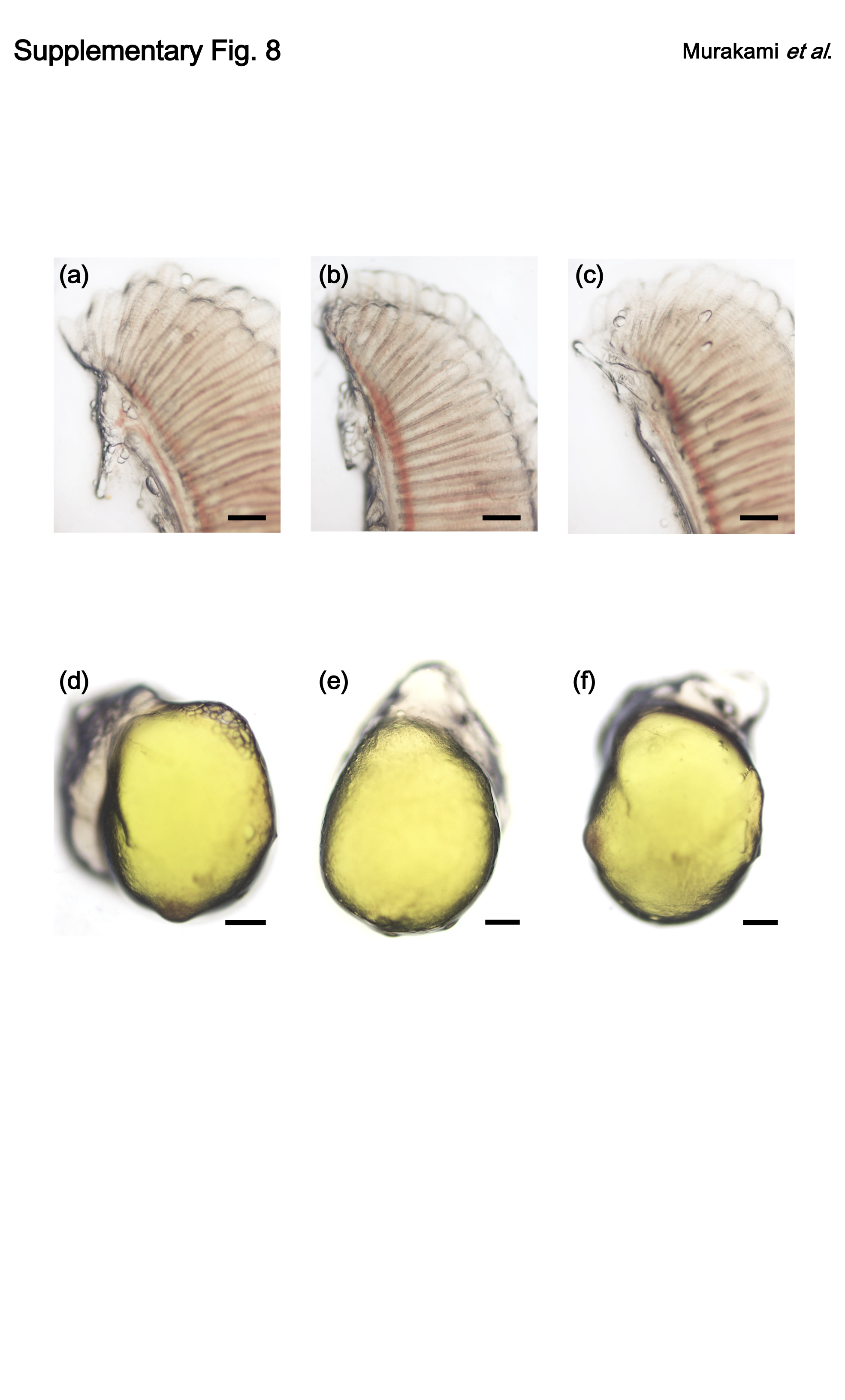

Supplement: Supplementary file 9 — Supplementary Figure 8. [file 41598_2022_22029_MOESM9_ESM.tiff]
