# Supplementary material for: Targeted deletion of ecto-5′-nucleotidase results in retention of inosine monophosphate content in postmortem muscle of medaka (Oryzias latipes)
Source: Sci Rep. 2022 Nov 3;12:18588. doi: 10.1038/s41598-022-22029-y (PMC9633828; doi:10.1038/s41598-022-22029-y)
Supplement: Supplementary file 10 — Supplementary Table 1. [file 41598_2022_22029_MOESM10_ESM.docx]

**Table S1.** Nt5ea and Nt5eb amino acid sequence identity and similarity across various species.

|  | | Amino acid similarity (%) | | | | | | | | | | | | | | | | | | |
| --- | --- | --- | --- | --- | --- | --- | --- | --- | --- | --- | --- | --- | --- | --- | --- | --- | --- | --- | --- | --- |
|  |  | 1 | 2 | 3 | 4 | 5 | 6 | 7 | 8 | 9 | 10 | 11 | 12 | 13 | 14 | 15 | 16 | 17 | 18 | 19 |
| 1. **Medaka Cab Nt5ea**  (ON_873732) | |  | 70.3 | 74.0 | 71.7 | 71.0 | 69.1 | 76.4 | 74.0 | 72.8 | 80.0 | 69.0 | 78.8 | 73.3 | 81.3 | 72.5 | 67.3 | 67.2 | 70.8 | 63.0 |
|  | 2. **Medaka Cab Nt5eb**  (ON_873733) | 58.3 |  | 73.8 | 74.1 | 72.9 | 67.9 | 72.5 | 75.0 | 73.4 | 72.6 | 77.5 | 70.8 | 80.9 | 71.9 | 81.5 | 73.6 | 66.7 | 69.4 | 63.0 |
|  | 3. **European eel**  (XP_035276486.1) | 62.6 | 64.4 |  | 77.6 | 78.6 | 72.3 | 76.9 | 82.0 | 80.7 | 77.7 | 72.1 | 73.4 | 75.2 | 75.5 | 76.5 | 70.1 | 67.8 | 72.1 | 65.0 |
|  | 4. **Common carp**  (XP_042633280.1) | 61.7 | 64.4 | 68.3 |  | 87.6 | 74.4 | 78.6 | 82.2 | 81.9 | 76.0 | 74.5 | 72.4 | 76.5 | 74.8 | 76.1 | 71.2 | 69.5 | 74.2 | 65.9 |
|  | 5. **Zebrafish**  (NP_957226.1) | 60.5 | 64.7 | 70.3 | 80.4 |  | 72.1 | 78.3 | 83.8 | 81.9 | 76.9 | 74.1 | 72.3 | 75.3 | 72.2 | 75.6 | 72.4 | 67.9 | 73.6 | 65.5 |
|  | 6. **Yellow catfish**  (XP_027032993) | 58.1 | 58.9 | 62.5 | 67.6 | 67.7 |  | 72.6 | 74.5 | 72.6 | 72.6 | 66.0 | 71.0 | 69.5 | 70.1 | 70.3 | 67.2 | 63.3 | 67.6 | 60.5 |
|  | 7. **Atlantic salmon**  (XP_013987183.2) | 65.6 | 61.4 | 66.7 | 66.9 | 68.6 | 60.5 |  | 78.9 | 78.0 | 80.5 | 71.8 | 77.4 | 75.4 | 80.9 | 75.5 | 71.3 | 68.1 | 71.7 | 63.8 |
|  | 8. **Atlantic salmon**  (XP_014060414.1) | 60.5 | 65.4 | 72.7 | 72.7 | 74.7 | 65.2 | 69.1 |  | 92.6 | 79.3 | 72.7 | 73.3 | 76.5 | 77.5 | 76.8 | 72.6 | 68.0 | 74.1 | 66.7 |
|  | 9. **Atlantic salmon**  (XP_013999620.1) | 59.9 | 63.8 | 71.4 | 71.9 | 73.3 | 63.9 | 67.6 | 90.0 |  | 78.0 | 71.0 | 72.8 | 74.9 | 75.9 | 75.5 | 72.0 | 66.6 | 72.9 | 64.9 |
|  | 10. **Tiger pufferfish**  (XP_003962750.3) | 71.3 | 60.5 | 64.5 | 64.8 | 65.7 | 60.0 | 70.1 | 65.7 | 65.4 |  | 73.2 | 82.6 | 75.1 | 87.7 | 74.7 | 70.4 | 68.6 | 72.4 | 64.3 |
|  | 11. **Tiger pufferfish**  (XP_029705660.1) | 57.2 | 66.6 | 61.5 | 62.6 | 64.3 | 55.3 | 58.0 | 61.2 | 59.8 | 58.7 |  | 70.2 | 80.4 | 74.4 | 77.9 | 77.2 | 67.9 | 70.2 | 62.8 |
|  | 12. **Nile tilapia**  (XP_019204379.1) | 70.5 | 59.0 | 61.9 | 61.8 | 62.3 | 59.8 | 66.5 | 62.2 | 62.1 | 51.7 | 56.5 |  | 72.8 | 85.8 | 71.9 | 68.2 | 67.8 | 69.2 | 61.7 |
|  | 13. **Nile tilapia**  (XP_003446719.1) | 61.5 | 71.0 | 65.0 | 66.1 | 66.0 | 58.1 | 63.0 | 65.9 | 63.9 | 72.5 | 69.3 | 60.5 |  | 75.9 | 84.2 | 77.3 | 69.9 | 71.4 | 64.6 |
|  | 14. **Greater amberjack**  (XP_022606637.1) | 73.7 | 60.5 | 64.2 | 64.0 | 65.2 | 58.7 | 72.1 | 64.9 | 63.7 | 63.1 | 60.1 | 77.5 | 63.7 |  | 75.0 | 70.7 | 68.6 | 72.7 | 64.5 |
|  | 15. **Greater amberjack**  (XP_022625844.1) | 59.8 | 74.0 | 67.1 | 66.5 | 68.0 | 61.2 | 64.2 | 68.9 | 66.6 | 79.6 | 69.6 | 61.5 | 77.9 | 63.0 |  | 75.9 | 68.0 | 70.9 | 63.5 |
|  | 16. **Tongue sole**  (XP_008311861.1) | 52.9 | 61.4 | 56.9 | 58.9 | 60.5 | 54.2 | 57.7 | 60.0 | 59.5 | 61.5 | 64.0 | 54.2 | 65.2 | 57.0 | 65.4 |  | 66.3 | 69.6 | 61.3 |
|  | 17. **Chicken**  (XP_040525077.1) | 54.0 | 55.4 | 56.2 | 58.4 | 58.0 | 50.8 | 55.7 | 56.0 | 55.0 | 55.8 | 55.4 | 53.6 | 56.9 | 54.1 | 55.6 | 52.2 |  | 73.1 | 66.4 |
|  | 18. **Cattle**  (NP_776554.2) | 58.2 | 57.6 | 60.6 | 69.5 | 63.5 | 58.0 | 60.4 | 62.5 | 61.5 | 53.6 | 57.3 | 57.2 | 58.7 | 58.5 | 59.7 | 56.3 | 60.9 |  | 85.2 |
|  | 19. **Human**  (NP_002517.1) | 50.3 | 51.4 | 72.1 | 65.9 | 55.5 | 51.3 | 52.5 | 55.7 | 55.2 | 57.8 | 50.6 | 51.3 | 52.2 | 52.0 | 53.1 | 49.1 | 53.8 | 81.4 |  |
|  |  | Amino acid identity (%) | | | | | | | | | | | | | | | | | | |
