# Supplementary material for: Targeted deletion of ecto-5′-nucleotidase results in retention of inosine monophosphate content in postmortem muscle of medaka (Oryzias latipes)
Source: Sci Rep. 2022 Nov 3;12:18588. doi: 10.1038/s41598-022-22029-y (PMC9633828; doi:10.1038/s41598-022-22029-y)
Supplement: Supplementary file 11 — Supplementary Table 2. [file 41598_2022_22029_MOESM11_ESM.docx]

**Table S2.** Results of microinjection and subsequent screening of founders.

| Targeted gene | Injected sgRNA | Injected embryos | Sexually matured | Germ-line transmission rate |
| --- | --- | --- | --- | --- |
| *nt5ea* | sgRNA-nt5ea-1 | 25 | 11 | 80.0% (4/5) |
| *nt5ea* | sgRNA-nt5ea-1 and sgRNA-nt5ea-2 | 53 | 21 | 28.6% (2/7) |
| *nt5eb* | sgRNA-nt5eb-1 | 19 | 10 | 66.7% (4/6) |
| *nt5eb* | sgRNA-nt5eb-1 and sgRNA-nt5eb-2 | 47 | 24 | 33.3% (3/9) |
