# Supplementary material for: Targeted deletion of ecto-5′-nucleotidase results in retention of inosine monophosphate content in postmortem muscle of medaka (Oryzias latipes)
Source: Sci Rep. 2022 Nov 3;12:18588. doi: 10.1038/s41598-022-22029-y (PMC9633828; doi:10.1038/s41598-022-22029-y)
Supplement: Supplementary file 12 — Supplementary Table 3. [file 41598_2022_22029_MOESM12_ESM.docx]

**Table S3.** Oligonucleotide sequences of the primers used in this study.

| Name | Sequence (5'-3') | Used for |
| --- | --- | --- |
| nt5ea-RT-FW | CATCGATTTAAAGCTATGACTCTCCGCTGGCGCTGCTGCG | Isolation of *nt5ea* |
| nt5ea-RT-RV | TCCACCGCTACCTCCATGATGATGATGATGATGCATGCAGAGGTTCAGGAAAAGACAC | Isolation of *nt5ea* |
| nt5eb-RT-FW | CATCGATTTAAAGCTATGGATGCGGTGCGCCCGGAGCGCA | Isolation of *nt5eb* |
| nt5eb-RT-RV | TCCACCGCTACCTCCATGATGATGATGATGATGCATGATTCCATGAAAACTCCAGAGC | Isolation of *nt5eb* |
| LUC-GFP-FW | CATCGATTTAAAGCTATGGAAGATGCCAAAAACAT | Construction of plasmid |
| LUC-GFP-RV | CCGCGGCCGCGAATTAAAAAACCTCCCACACCTCC | Construction of plasmid |
| Backbone1-FW | AATTCGCGGCCGCGGCGCCAATGCATTGGGCCCGG | Construction of plasmid |
| Backbone1-RV | AGCTTTAAATCGATGGGATCCTGCAAAAAG | Construction of plasmid |
| Backbone2-FW | GGAGGTAGCGGTGGAACTACTAGTATGGAAGATGCCAAAAACATTAAG | Construction of plasmid |
| OligoA-nt5ea-Target1 | CTAATACGACTCACTATAGCAGGATGTTCACCAAAGTGAGTTTTAGAGCTAGAAATAGCA | Synthesis of sgRNA-nt5ea-1 |
| OligoA-nt5ea-Target2 | CTAATACGACTCACTATAGGTGATGCGGCCTTCCACTGCGTTTTAGAGCTAGAAATAGCA | Synthesis of sgRNA-nt5ea-2 |
| nt5ea-HMA1-FW | GTCCGGCTTCGAGCTGACTCTGCTCCACACC | HMA analysis for *nt5ea* |
| nt5ea-HMA1-RV | CCAAGTTTGTTCATGAAATGCGCA | HMA analysis for *nt5ea* |
| nt5ea-HMA2-FW | AGGTTTAGACAGAAAACAAGTTTAGAGC | HMA analysis for *nt5ea* |
| nt5ea-HMA2-RV | GCAGAGGTTCAGGAAAAGACACAGACTC | HMA analysis for *nt5ea* |
| nt5ea-Large1-FW | ACCTGAATGGAACGTTTGAAGAGTG | Detection of large deletion at *nt5ea* |

**Table S3.** *continues*.

| nt5ea-Large1-RV | CGTTTGTACTGCGTTCATCGATTGT | Detection of large deletion at *nt5ea* |
| --- | --- | --- |
| nt5ea-Large2-FW | GCGCTGCTGCGCCCTCGGCGCCTTG | Detection of large deletion at *nt5ea* |
| nt5ea-Large2-RV | CTGAACCTGTAGAGCTTCTCCGTTG | Detection of large deletion at *nt5ea* |
| OligoA-nt5eb-Target1 | CTAATACGACTCACTATAGCGGGGTGGCGCGGAGAGCCAGTTTTAGAGCTAGAAATAGCA | Synthesis of sgRNA-nt5eb-1 |
| OligoA-nt5eb-Target2 | CTAATACGACTCACTATAGTAGAGGGACGCATTAAAGTCGTTTTAGAGCTAGAAATAGCA | Synthesis of sgRNA-nt5eb-2 |
| nt5eb-HMA1-FW | GCCCTGTTCTCCTCATCCTCTC | HMA analysis for *nt5eb* and detection of large deletion at *nt5eb* |
| nt5eb-HMA1-RV | CATGGCGTCATATCGCAGCAAGTTC | HMA analysis for *nt5eb* |
| nt5eb-HMA2-FW | TCGTGACTGGAGGAGATGGATTTAG | HMA analysis for *nt5eb* |
| nt5eb-HMA2-RV | CATGATTCCATGAAAACTCCAG | HMA analysis for *nt5eb* and detection of large deletion at *nt5eb* |
| nt5eb-Large1-FW | GTGACCTTTGATGAGAACGGGAACG | Detection of large deletion at *nt5eb* |
| nt5eb-Large1-RV | CTGGGATGAGTAATTAGCCAGAC | Detection of large deletion at *nt5eb* |
